# Per‐ and Polyfluoroalkyl Substances (PFAS) Exposure Profiles and Their Predictors in a Study of US Volunteer Firefighters

**DOI:** 10.1002/ajim.70104

**Published:** 2026-06-14

**Authors:** Katherine A. Lubina, Zorimar Rivera‐Núñez, Miriam M. Calkins, Robert Laumbach, Shou‐En Lu, Jefferey L. Burgess, Elena Austin, Derrick L. Edwards, Maria D. H. Koeppel, Jaclyn M. Goodrich, Zhihua (Tina) Fan, Chang Ho Yu, Kaleigh M. Hinton, Casey C. Grant, Brian Kubiel, Daniel Roy, Judith M. Graber

**Affiliations:** ^1^ Rutgers, the State University of New Jersey School of Public Health Piscataway New Jersey USA; ^2^ Rutgers, the State University of New Jersey Environmental and Occupational Health Science Institute Piscataway New Jersey USA; ^3^ National Institute for Occupational Safety and Health Centers for Disease Control and Prevention Cincinnati Ohio USA; ^4^ University of Arizona Mel and Enid Zuckerman College of Public Health Tucson Arizona USA; ^5^ University of Washington, Hans Rosling Center for Population Health Seattle Washington USA; ^6^ Tennessee Tech University, Counseling & Psychology Cookeville Tennessee USA; ^7^ Center for Fire, Rescue, & EMS Health Research NDRI‐USA Leawood Kansas USA; ^8^ University of Michigan School of Public Health Ann Arbor Michigan USA; ^9^ New Jersey Department of Health Public Health and Environmental Laboratories Ewing New Jersey USA; ^10^ Gillings School of Global Public Health, Department of Epidemiology Chapel Hill North Carolina USA; ^11^ DSRAE, LLC Belmont Massachusetts USA; ^12^ Toms River Fire Department Toms River New Jersey USA; ^13^ Monmouth Fire Department Monmouth Maine USA

**Keywords:** first responders, occupational exposure, per‐ and polyfluoroalkyl substances (PFAS), volunteer firefighters

## Abstract

**Introduction:**

Firefighters may experience occupational exposure to per‐ and polyfluoroalkyl substances (PFAS). Volunteer firefighters make up 65% of the US fire service, but their serum PFAS profiles have not been well characterized. This study aims to (1) describe PFAS serum profiles among US volunteer firefighters from 9 states enrolled in the Firefighter Cancer Assessment and Prevention Study (CAPS), and (2) identify predictors of their serum PFAS concentrations.

**Methods:**

Twelve PFAS were quantified in serum of 550 active volunteer firefighters collected at enrollment into CAPS between 2020 and 2023. Fire service, occupational, sociodemographic, and health predictors of interest were captured in the CAPS enrollment survey. PFAS detection frequencies, average concentrations, and distributions were characterized overall and by regional groups. Associations between serum PFAS and predictors of interest were assessed using multivariable generalized estimating equation models for all participants and by sex in those < 50 years old.

**Results:**

CAPS participants' serum PFAS profiles varied by geography, age, and sex. Firefighting, occupational, sociodemographic, and health factors were associated with serum PFAS in the full cohort. In sex‐stratified models, some associations differed between males and females and from the full cohort.

**Conclusion:**

Stronger associations between sociodemographic and health factors with serum PFAS, with less strong associations between serum PFAS and occupational and fire service factors highlight the likely multifaceted exposure experience of volunteer firefighters and the relative importance of community factors. Sex and age differences in serum profiles and associations with predictors may imply different exposure and elimination pathways among male and female volunteer firefighters.

## 1 Introduction

Per‐ and polyfluoroalkyl substances (PFAS) include thousands of anthropogenic chemicals containing highly stable carbon‐fluorine bonds [1, 2]. Despite their widespread use in industrial and consumer applications, only a handful of these chemicals have been systematically studied [3, 4]. PFAS are of public health concern because several long‐chain PFAS are detected in the blood of nearly all US residents and are associated with numerous adverse health outcomes including lipid dysregulation, increased incidence of certain cancers, immune modulation, and endocrine disruption [5, 6, 7]. Among general populations, ingestion of contaminated water and food, inhalation of particles, and dermal absorption of PFAS‐containing products are thought to be the primary PFAS exposure pathways [5]. However, the relative importance of these exposure pathways varies by physiological, geographic, and temporal factors [8]. Some populations such as firefighters may be additionally exposed to PFAS through their occupations [9, 10].

Firefighters may be exposed to PFAS through use of class‐B aqueous film‐forming firefighting foams (AFFF), combustion products, and possibly their firefighter protective clothing (turnout gear) [11, 12, 13, 14, 15]. Firefighters have been found to have higher than average serum concentrations of several PFAS compounds (including perfluorooctanoic acid [PFOA] [13, 16, 17], perfluorooctane sulfonic acid [PFOS] [16, 17, 18], perfluorohexane sulfonic acid [PFHxS] [15, 16, 17, 18, 19, 20], and perfluorononanoic acid [PFNA] [13, 15, 16]) compared to reference populations. However, there is heterogeneity in the concentrations of PFAS compounds reported in firefighter biomonitoring studies, which may be attributable to multiple factors such as changes in exposure over time or by geographic, individual, and physiological factors [21, 22, 23].

Of the over one million firefighters in the United States, 65% are volunteers [24]. However, despite volunteer firefighters having the same responsibilities as career firefighters [24], few studies have assessed PFAS serum profiles in volunteer firefighters. While volunteer firefighters anecdotally report less frequent AFFF use and may spend less time at their fire stations than career firefighters, they may have other PFAS exposure pathways. Volunteer firefighters tend to be under‐resourced compared with career firefighters [25]. For example, their turnout gear may be older or fit poorly, opening potential fire scene and gear‐related exposure pathways [11]. Many volunteer fire departments do not have gear (extraction) washers or showers in the fire station [26]. Many volunteer firefighters do not have a second set of turnout gear to use while the first one is being cleaned, thus their gear may be laundered less frequently. Unlike many career fire stations, volunteer fire stations may not be equipped with separate storage areas for gear or equipment that may off‐gas PFAS or generate dust containing PFAS. Volunteer fire departments may not have, or may not regularly update, standard operating procedures for post‐contamination exposure reduction. Volunteer firefighters may also use personal vehicles to transport themselves or their gear prior to complete decontamination. Volunteer firefighters may perform multiple or overlapping duties at a fire scene when few members respond to a call, possibly increasing the number of hours spent on scene. These factors can lead to a build‐up of contaminants and prolong potential exposure.

Volunteer firefighters tend to live in the communities they serve. Volunteer firefighters typically serve rural and suburban communities, with 95% of volunteer firefighters serving communities of 25,000 or fewer people [24]. Most volunteer firefighters hold full or part time jobs in addition to their volunteer firefighting duties [27]. Thus their sociodemographic, occupational, and other community‐related characteristics may be as important as their firefighting experiences in understanding their PFAS exposures.

Characterization of predictors of serum PFAS concentrations in volunteer firefighters is needed to elucidate community and firefighting‐related exposure sources and could inform evidence‐based exposure mitigation and reduction strategies. The objectives of this study are to (1) describe PFAS serum profiles among US volunteer firefighters enrolled in the Firefighter Cancer Assessment and Prevention Study (CAPS), and (2) identify predictors of their serum PFAS concentrations.

## 2 Materials and Methods

### 2.1 Study Design and Participants

To address the study aims, we used cross‐sectional biological samples paired with survey data, which were collected from participants at enrollment in a large longitudinal cohort study. CAPS was designed to examine cancer risk factors and prevention behaviors among volunteer firefighters, and is a research partner of the national Fire Fighter Cancer Cohort Study (FFCCS) [27, 28]. Between 2020 and 2023, CAPS enrolled 607 volunteer firefighters over the age of 18 from 41 fire departments in nine US states—Connecticut (CT), Kansas (KS), Illinois (IL), Maryland (MD), Maine (ME), Missouri (MO), New Jersey (NJ), Tennessee (TN), and Washington (WA). Researchers used a referral sampling approach to identify volunteer or combination fire departments interested in participating. Volunteer firefighters from these 41 departments were then invited to participate in the study. Participants provided written informed consent at enrollment. Institutional Review Boards at the University of Miami, the University of Arizona, and Rutgers, the State University of New Jersey approved this research project (STUDY00002192, Pro2019000811).

### 2.2 CAPS PFAS Measures

Twelve PFAS were quantified in CAPS participants' serum by the New Jersey Department of Health Public Health and Environmental Laboratories (NJDOH PHEL). Trained research staff collected blood samples in 10 mL serum separator tubes at study enrollment events at the fire stations. Samples were stored at −80°C until shipped on dry ice to NJDOH PHEL for PFAS quantification. PFOA, PFOS, PFHxS, PFNA, perfluorodecanoic acid (PFDA), perfluoroundecanoic acid (PFUnDA), 2‐(n‐methyl‐perfluorooctane sulfonamido) acetic acid (MeFOSAA), perfluoroheptanoic acid (PFHpA), perfluorooctane sulfonamide (FOSA), perfluorobutane sulfonic acid (PFBS), perfluorododecanoic acid (PFDoDA), and 2‐(n‐ethyl‐perfluorooctane sulfonamido) acetic acid (EtFOSAA) were quantified in serum by an optimized CDC method (No. 6304.4) using high performance liquid chromatography‐online solid phase extraction‐tandem mass spectrometry (HPLC‐SPE MS/MS) [29]. The limit of detection (LOD) varied by compound and was used to determine the detection frequency (DF).

### 2.3 CAPS Survey Measures: Predictors of Interest

Most enrollment surveys were self‐administered at the time of blood draw with a small proportion administered by phone interview. The survey included questions about sociodemographic characteristics, health behaviors, firefighting experience, and employment history. The CAPS survey was administered through the Rutgers REDCap system, a secure survey platform [30, 31]. A literature review and expert knowledge‐informed selection of predictors in the domains below. For details on variable categorization, see Table S1.

- *Fire service factors*: years of volunteer fire service; career firefighter status; average number of monthly calls; average number of monthly hours spent responding to calls, training, and at the fire station for non‐response duties (e.g., meetings); current volunteer rank; years in current rank; tasks performed on fire calls.
- *Occupational factors*: employment status; current primary occupation [32]; currently work > 1 job; any history of active‐duty military service; time since military service; participation in the 9/11/2001 World Trade Center (WTC) disaster response.
- *Demographics and health/health behaviors*: age in years; sex at birth; race and ethnicity; highest level of education; annual household income; body mass index (BMI) [33]; cigarette smoking; smokeless tobacco (SLT) use; hazardous drinking behavior based on AUDIT‐C score [34, 35]; home residence located within 5 miles of a military base or airport.

Fire departments were combined into regions, with a minimum sample size of 20 individuals per region. Regions were created by state for CT, KS and MO (combined), MD, TN, and WA, and sub‐state regions were created for central ME, southern ME, north NJ, central NJ, and coastal NJ. Two departments were excluded from regional groupings due to geographic isolation from other departments and insufficient participants to analyze separately (< 20 participants). These two departments were included in all other analyses.

### 2.4 Statistical Analysis

Sociodemographic, health, occupational and firefighting characteristics of participating firefighters were summarized using descriptive statistics for the full analytic population, participants under 50, and participants under 50 stratified by sex. Categories with fewer than 10 participants were suppressed or combined due to privacy considerations. For each of the 12 PFAS measured, the DF, unadjusted geometric mean (GM), 95% confidence interval (95% CI), and percentiles were calculated for all CAPS participants. We also calculated PFAS distributions in CAPS participants stratified by age (under 50 years old), sex, and region. For sex‐stratified PFAS distributions, we restricted the age of participants to those under age 50, as there are differences between pre‐ and post‐menopausal women's serum PFAS concentrations due to changes in elimination pathways [36]. Age 50 was selected as a reasonable cutoff for menopause [37] and there are positive associations between age and many PFAS measured in this study [7, 38, 39]. As 90% of female firefighters in our study were under age 50, we did not conduct the sex‐stratified analyses in the older age group. PFAS detected above the LOD in at least 70% of participants (high‐detect compounds) were analyzed as continuous variables. Measures below the LOD were imputed using LOD divided by the square root of two (LOD/√2) [40]. High‐detect compounds were naturallog (ln) transformed for modeling to satisfy distributional assumptions [41]. Compounds detected in fewer than 70% of participants (low‐detect) were dichotomized as above and below the LOD. Correlations and regressions were used to evaluate bivariate relationships between PFAS and predictors of interest.

Of the predictors described, missing data ranged from 0% to 17%. Multiple imputation was done to create 20 imputed datasets using the fully conditional specification (FCS) algorithm in SAS v9.4. FCS has the ability to specify multiple estimation models for continuous, categorical and binary variables [42]. Auxiliary variables used in the imputation models included age, sex at birth, military service, and fire department, all of which had no missing data. Distributions, bivariate, and multivariable results were compared between imputed and non‐imputed data for all imputed variables and were similar (results not shown).

Latent class analysis (LCA) was used as a dimension reduction approach using the 27 non‐mutually exclusive firefighting tasks participants reported performing on calls, using the R package poLCA [43]. These firefighting tasks are described in Table S1. LCA uses probabilistic unsupervised clustering to identify underlying population subgroups within a heterogeneous population [44, 45]. Models of one to six classes were fit and compared using fit and performance statistics (AIC, BIC) to select the optimal model (e.g., k vs. k – 1 classes). Relevance and ease of interpretation were also considered. Distributions of firefighting task variables were examined within assigned categories to review the appropriateness of individuals' class assignments. For example, if a firefighter who reported many technical tasks was classified in the command category, this may indicate poor fit. Firefighting task classes were then used in regression models to assess the associations between firefighting task classes and serum PFAS profiles. Individual firefighting task variables (i.e., performing vs. not performing each task) were also examined in regression models to identify whether individual tasks may have been driving associations between serum PFAS profiles and a particular class.

Generalized estimating equation (GEE) modeling was used to assess the population‐level associations between the predictors of interest and each serum PFAS while accounting for the likely correlation of individuals within fire departments. Fire department was used as the clustering variable. An exchangeable correlation structure was selected over independent, autoregressive and unstructured correlation structures based on lower QIC values [46]. In this exploratory analysis, adjusted models included a covariate set selected based on trends observed in bivariate analyses (*p* ≤ 0.20). We also considered which covariates best characterized the factors of interest within domains (i.e., in our primary models, the current primary occupation variable was included instead of the employment status variable as it better characterized participants' current occupations vs. full‐time, part‐time, or no employment). The same covariate set was regressed on each PFAS for model comparability: age, sex, education, household income, hazardous drinking, cigarette use, pesticide or herbicide use, active‐duty military service, current primary occupation, years of volunteer firefighting experience, career firefighter status, average monthly calls, current rank, years in current rank, and firefighting task LCA class. Nine GEE models were implemented. PFOA, PFOS, PFNA, PFHxS, PFDA, and PFUnDA were modeled as ln‐transformed continuous outcomes, and MeFOSAA, PFHpA, and FOSA were modeled as detected versus not detected binary outcomes. GEE model results were not presented for PFBS, PFDoDA, and EtFOSAA because a high percentage (> 85%) of values were below LOD, which led to problems of model convergence.

Due to sex differences in serum PFAS concentrations observed in our data and reported in other studies [7, 16, 39, 47], we also conducted sex‐stratified multivariable GEE modeling among male and female volunteer firefighters under age 50. However, because of reduced sample size and resulting model convergence, a reduced covariate set was selected for these models. Also, due to sample size considerations, categories for some of the variables were combined. In addition, only sex‐stratified models for high‐detect PFAS are presented, due to model instability and non‐convergence for low‐detect PFAS, even after reducing the covariate set. Variables included in sex‐stratified models included: age, education (≤ high school, some college, ≥bachelor's degree), annual household income (<$50,000, $50,000‐ < $100,000, ≥$100,000), hazardous drinking, cigarette use, pesticide or herbicide use, employed ≥ 35 h/week (versus not), years of volunteer firefighting experience, ever (vs. never) a career firefighter, average number of monthly calls (< 5, 5 – 14, ≥ 15).

Data management, descriptive, multiple imputation, and GEE analyses were conducted with SAS v. 9.4. Data visualization and LCA were done in R/RStudio v. 2024.04.2 + 764 or later.

## 3 Results

Of the 607 CAPS enrollees, 14 did not complete an enrollment survey, 39 did not provide a blood serum sample for PFAS quantification, and four were inactive (retired) firefighters (Figure S1). Of these 550 active volunteer firefighters, 360 were under age 50.

This population of 550 active volunteer firefighters was mostly non‐Hispanic White (90.2%) and male (88.9%) (Table 1). Three‐quarters of participants completed some post‐secondary education. Over one‐third screened positive for hazardous drinking. About 10% were current cigarette smokers and 11.1% were current SLT users. Most participants (85.5%) were employed, with significant proportions working in public administration (17.4%) and construction‐related jobs (15.9%). The majority (71.9%) of those with active‐duty military experience reported serving more than 15 years ago. Further, over 80% reported less than one year of active‐duty military service (data not shown). Hence, time since active duty and years of active duty were not explored further.

**Table 1:** Sociodemographic, health, occupational, and firefighting characteristics of Firefighter Cancer Assessment and Prevention Study (CAPS) participants (*n* = 550).

	All (*n* = 550)		Age < 50 (*n* = 360)	
	*n*	% (95% CI)	*n*	% (95% CI)
Demographic and health characteristics				
Age (years, mean (95% CI))	550	42.7 (41.5, 44.0)	360	33.5 (32.5, 34.4)
Male	489	88.9 (86.3, 91.5)	306	85.0 (81.3, 88.7)
Non‐Hispanic white (*n* = 548)	494	90.1 (87.6, 92.6)	313	86.9 (83.4, 90.4)
BMI^a^				
Healthy and underweight (< 25.0 kg/m^2^)	106	19.3 (16.0, 22.6)	77	21.4 (17.1, 25.6)
Overweight (25.0–29.9 kg/m^2^)	212	38.5 (34.5, 42.6)	136	37.8 (32.7, 42.8)
Obesity (≥ 30 kg/m^2^)	232	42.2 (38.0, 46.3)	147	40.8 (35.7, 45.9)
Education (*n* = 549)				
High school or below	141	25.7 (22.0, 29.3)	90	25.1 (20.6, 29.6)
Some college, associates, technical	230	41.9 (37.8, 46.0)	158	44.0 (38.9, 49.2)
Bachelor's degree or above	178	32.4 (28.5, 36.4)	111	30.9 (26.1, 35.7)
Annual household income (*n* = 515)				
< $50,000	121	23.5 (19.8, 27.2)	89	26.3 (21.6, 31.1)
$50,000–< $75,000	83	16.1 (12.9, 19.3)	64	18.9 (14.7, 23.1)
$75,000–< $100,000	100	19.4 (16.0, 22.8)	57	16.9 (12.9, 20.9)
$100,000–< $150,000	111	21.6 (18.0, 25.1)	67	19.8 (15.6, 24.1)
> $150,000	100	19.4 (16.0, 22.8)	61	18.0 (13.9, 22.2)
WTC Present (*n* = 549)^b^	30	5.5 (3.6, 7.4)	< 10	1.7 (0.3, 3.0)
Pesticide/herbicide use in past year (*n* = 525)	256	48.8 (44.5, 53.1)	145	42.8 (37.5, 48.1)
Cigarette use (*n* = 536)				
Never	404	75.4 (71.7, 79.0)	267	76.5 (72.0, 81.0)
Former	79	14.7 (11.7, 17.7)	43	12.3 (8.9, 15.8)
Current	53	9.9 (7.4, 12.4)	39	11.2 (7.9, 14.5)
Smokeless tobacco use (*n* = 477)				
Never	372	78.0 (74.3, 81.7)	242	74.0 (69.2, 78.8)
Former	52	10.9 (8.1, 13.7)	38	11.6 (8.1, 15.1)
Current	53	11.1 (8.3, 13.9)	47	14.4 (10.6, 18.2)
Hazardous drinking (*n* = 548)^c^	206	37.6 (33.5, 41.7)	141	39.2 (34.1, 44.2)
Home < 5 miles of military base/airport (*n* = 536)	93	17.4 (14.1, 20.6)	64	18.2 (14.2, 22.3)
Occupational characteristics				
Military service^d^	61	11.1 (8.5, 13.7)	29	8.1 (5.2, 10.9)
Employment status (*n* = 532)				
Employed full time (≥ 35 h/week)	404	75.9 (72.3, 79.6)	291	84.6 (80.8, 88.4)
Employed part time (< 35 h/week)	51	9.6 (7.1, 12.1)	29	8.4 (5.5, 11.4)
Not employed	77	14.5 (11.5, 17.5)	24	7.0 (4.3, 9.7)
More than 1 job (*n* = 531)	99	18.6 (15.3, 22.0)	73	21.3 (17.0, 25.7)
Current occupation categories (*n* = 522)^e^				
Construction/Ag./Mining/Building services	83	15.9 (12.8, 19.0)	57	16.8 (12.8, 20.8)
Fire Protection	36	6.9 (4.7, 9.1)	33	9.7 (6.5, 12.9)
Professional, Finance, Education, Info.	65	12.5 (9.6, 15.3)	46	13.5 (9.9, 17.2)
Public Admin., Justice/Public Order	91	17.4 (14.2, 20.7)	52	15.3 (11.4, 19.1)
Retail, Health Care, Arts/Hospitality	62	11.9 (9.1, 14.7)	51	15.0 (11.2, 18.8)
Other Services	57	10.9 (8.2, 13.6)	41	12.1 (8.6, 15.5)
Transportation, Wholesale, Manufacturing	51	9.8 (7.2, 12.3)	36	10.6 (7.3, 13.9)
Not employed	77	14.8 (11.7, 17.8)	24	7.1 (4.3, 9.8)
Firefighting (FF) characteristics				
Volunteer FF experience (years, mean (95% CI))	548	17.5 (16.2, 18.8)	358	10.0 (9.1, 10.9)
Current volunteer rank (*n* = 549)				
Firefighter (incl. investigators, instructors)	234	42.6 (38.5, 46.8)	179	49.9 (44.7, 55.1)
Firefighter/EMT	86	15.7 (12.6, 18.7)	70	19.5 (15.4, 23.6)
Driver Operator/Engineer	24	4.4 (2.7, 6.1)	< 10	< 5
Officers	100	18.2 (15.0, 21.5)	59	16.4 (12.6, 20.3)
All Chiefs	71	12.9 (10.1, 15.7)	26	7.2 (4.5, 9.9)
Auxiliary + Recruit	34	6.2 (4.2, 8.2)	> 10	< 5
Years in current volunteer FF rank (*n* = 549)				
0−1 years	156	28.4 (24.6, 32.2)	125	34.8 (29.9, 39.8)
2–4 years	157	28.6 (24.8, 32.4)	114	31.8 (26.9, 36.6)
5−10 years	118	21.5 (18.0, 24.9)	73	20.3 (16.2, 24.5)
> 10 years	118	21.5 (18.0, 24.9)	47	13.1 (9.6, 16.6)
Average monthly FF calls (*n* = 484)				
< 5 calls	127	26.2 (22.3, 30.2)	85	25.5 (20.8, 30.2)
5−10 calls	147	30.4 (26.3, 34.5)	102	30.6 (25.7, 35.6)
11−20 calls	122	25.2 (21.3, 29.1)	79	23.7 (19.1, 28.3)
> 20 calls	88	18.2 (14.7, 21.6)	67	20.1 (15.8, 24.4)
Career FF status (*n* = 549)^f^				
Never	452	82.3 (79.1, 85.5)	301	83.8 (80.0, 87.7)
Current	64	11.7 (9.0, 14.4)	> 40	> 10
Former	33	6.0 (4.0, 8.0)	< 10	< 5
FF task classes (*n* = 536)^g^				
Support	155	28.9 (25.1, 32.8)	76	21.8 (17.5, 26.2)
Technical	62	11.6 (8.9, 14.3)	44	12.6 (9.1, 16.2)
General	154	28.7 (24.9, 32.6)	107	30.7 (25.9, 35.6)
Interior, medical	70	13.1 (10.2, 15.9)	59	17.0 (13.0, 20.9)
Some on‐scene	95	17.7 (14.5, 21.0)	62	17.8 (13.8, 21.9)
State/regional group (# of depts.)				
Connecticut (2)	29	5.3 (3.4, 7.2)	17	4.8 (2.5, 7.0)
Kansas and Missouri (6)	52	9.5 (7.0, 12.0)	37	10.4 (7.2, 13.5)
Maryland (2)	32	5.9 (3.9, 7.8)	19	5.3 (3.0, 7.7)
Maine—Central (3)	39	7.1 (5.0, 9.3)	22	6.2 (3.7, 8.7)
Maine—Southern (14)	45	8.2 (5.9, 10.5)	27	7.6 (4.8, 10.3)
New Jersey—Central (7)	50	9.1 (6.7, 11.6)	38	10.6 (7.4, 13.9)
New Jersey—Coastal (2)	110	20.1 (16.7, 23.5)	56	15.7 (11.9, 19.5)
New Jersey—North (4)	60	11.0 (8.3, 13.6)	35	9.8 (6.7, 12.9)
Tennessee (16)	74	13.5 (10.7, 16.4)	54	15.1 (11.4, 18.9)
Washington (5)	51	9.3 (6.9, 11.8)	47	13.2 (9.6, 16.7)

*Note:* Cells with fewer than 10 participants were suppressed for privacy reasons.

^a^ BMI: Body mass index, calculated using the equation weight (lb)/[height (in)]^2^ × (703 (kg/m^2^)/(lb/in^2^)) [34].

^b^ WTC: World Trade Center rescue and recovery efforts following 9/11/2001.

^c^ Hazardous drinking based on AUDITC score (≥ 3 for females and ≥ 4 for males) [35, 36].

^d^ Military service includes current or former active duty in US military, beyond basic training.

^e^ Current primary occupation categories were created from survey responses using the North American Industry Classification System (NAICS) [33]. Ag: Agriculture contains forestry and fishing industry occupations. Hospitality includes entertainment, accommodation, and recreation industry occupations.

^f^ All participants were current volunteer firefighters. Some were concurrently career firefighters in addition to their volunteer duties, and some were former career firefighters.

^g^ FF task classes created using latent class analysis.

On average, this cohort of volunteer firefighters had 17.5 years of volunteer firefighting experience. Under half (43.0%) had been in their current rank for five or more years. The most common rank was firefighter (42.6%), with fewer serving as firefighter‐EMTs, officers, and chiefs (15.7%, 18.2% and 12.9%, respectively). Nearly 18% ever worked as a career firefighter, of whom 43.3% worked as a career firefighter for five years or less.

Firefighters under age 50 generally had similar sociodemographic, health, fire service, and occupational characteristics as the full cohort (Table 1). A smaller proportion of those under 50 were not employed compared to those 50 and over (7% vs. 14.5%). There were some sex differences in these characteristics among those under 50 (Table S2). Compared to male volunteer firefighters under 50, females had lower BMIs and fewer used smokeless tobacco (never users: > 80% females, 70.5% males). No female firefighters under age 50 had a history of military service, and on average had fewer years of volunteer firefighting experience compared to their male counterparts (6.9 vs. 10.5 years, respectively).

Using LCA, five firefighting task classes were created (Table S3 and Figure S2). Class 1 (support) included firefighters who reported tasks such as command, staging, traffic control, and recruit/probationary. Firefighters in class 2 (technical) performed specialized and on‐scene structural response tasks, such as technical rescue, hazmat, fire attack, ventilation, overhaul. Class 3 (general) performed interior and exterior tasks such as driver, pump panel, interior, and forcible entry. All members of class 4 (interior and medical) performed interior, medical/EMS, fire attack, and fire suppression. Class 5 (some on‐scene) had fewer participants performing most tasks than other classes. Nearly 30% of participants were placed in classes 1 and 3, with 11.6%, 13.1%, and 17.7% in classes 2, 4, and 5 (Table 1).

CAPS volunteer firefighters' serum PFAS GMs ranged from 0.03 ng/mL for PFUnDA to 2.87 ng/mL for PFOS for the high‐detect PFAS (Table 2). Firefighters under 50 had lower GM PFAS compared with those 50 and older for all 6 high‐detect PFAS (*p* < 0.01). There were also notable sex differences in serum PFAS concentrations, with females having lower PFOA, PFOS, PFNA, and PFHxS than males (*p* < 0.001). DFs were similar across groups.

**Table 2:** Detection frequency (DF), geometric means (GM), and 95% confidence intervals (95%CI) of serum PFAS measures among active CAPS volunteer firefighters (*n* = 550), CAPS < 50 years (*n* = 360), and sex‐stratified among CAPS < 50 years (Males *n* = 306, Females *n* = 54).

PFAS	Abbr.	Population	Detection frequency (DF)		PFAS distributions						
			*n* > LOD^a^	% (95% CI)	GM (95% CI)	*p*‐value^b^	Min	25th	Median	75th	Max
Perfluorooctanoic acid	PFOA	All CAPS	549	99.8 (99.5, 100.0)	1.28 (1.21, 1.36)	0.0001	< LOD	0.83	1.32	1.96	9.25
		CAPS < 50 years	359	99.7 (99.2, 100.0)	1.18 (1.10, 1.27)		< LOD	0.77	1.17	1.83	9.25
		Males < 50 years	306	100.0 (100.0, 100.0)	1.28 (1.19, 1.37)	0.0002	0.17	0.85	1.25	1.90	9.25
		Females < 50 years	53	98.1 (94.4, 100.0)	0.77 (0.61, 0.98)		< LOD	0.50	0.77	1.30	3.27
Perfluorooctane sulfonic acid	PFOS	All CAPS	549	99.8 (99.5, 100.0)	2.87 (2.70, 3.05)	< 0.0001	< LOD	1.79	2.94	4.56	52.80
		CAPS < 50 years	359	99.7 (99.2, 100.0)	2.49 (2.31, 2.68)		< LOD	1.63	2.57	3.89	52.80
		Males < 50 years	306	100.0 (100.0, 100.0)	2.69 (2.50, 2.88)	0.0008	0.40	1.73	2.69	4.13	21.40
		Females < 50 years	53	98.1 (94.4, 100.0)	1.60 (1.21, 2.13)		< LOD	0.98	1.65	2.69	52.80
Perfluorononanoic acid	PFNA	All CAPS	549	99.8 (99.5, 100.0)	0.56 (0.54, 0.59)	< 0.0001	< LOD	0.40	0.59	0.80	35.20
		CAPS < 50 years	359	99.7 (99.2, 100.0)	0.52 (0.49, 0.55)		< LOD	0.37	0.54	0.75	11.30
		Males < 50 years	306	100.0 (100.0, 100.0)	0.56 (0.53, 0.59)	0.0004	0.09	0.41	0.56	0.77	11.30
		Females < 50 years	53	98.1 (94.4, 100.0)	0.36 (0.28, 0.45)		< LOD	0.26	0.33	0.60	1.93
Perfluorohexane sulfonic acid	PFHxS	All CAPS	549	99.8 (99.5, 100.0)	1.17 (1.09, 1.25)	< 0.0001	< LOD	0.74	1.23	1.99	26.67
		CAPS < 50 years	359	99.7 (99.2, 100.0)	1.06 (0.97, 1.15)		< LOD	0.65	1.13	1.80	5.82
		Males < 50 years	306	100.0 (100.0, 100.0)	1.19 (1.10, 1.29)	< 0.0001	0.14	0.77	1.24	1.95	5.82
		Females < 50 years	53	98.1 (94.4, 100.0)	0.55 (0.43, 0.71)		< LOD	0.34	0.60	0.90	3.15
Perfluorodecanoic acid	PFDA	All CAPS	423	76.9 (73.4, 80.4)	0.07 (0.06, 0.07)	0.0097	< LOD	0.02	0.08	0.16	8.76
		CAPS < 50 years	271	75.3 (70.8, 79.8)	0.06 (0.05, 0.07)		< LOD	0.02	0.07	0.15	8.76
		Males < 50 years	232	75.8 (71.0, 80.6)	0.06 (0.06, 0.07)	0.2402	< LOD	0.02	0.07	0.15	8.76
		Females < 50 years	39	72.2 (59.9, 84.6)	0.05 (0.04, 0.07)		< LOD	< LOD	0.05	0.14	1.16
Perfluoroundecanoic acid	PFUnDA	All CAPS	422	76.7 (73.2, 80.3)	0.03 (0.03, 0.04)	0.0001	< LOD	0.01	0.05	0.10	0.82
		CAPS < 50 years	266	73.9 (69.3, 78.4)	0.03 (0.02, 0.03)		< LOD	< LOD	0.04	0.09	0.66
		Males < 50 years	227	74.2 (69.3, 79.1)	0.03 (0.03, 0.03)	0.6423	< LOD	< LOD	0.04	0.09	0.66
		Females < 50 years	39	72.2 (59.9, 84.6)	0.03 (0.02, 0.04)		< LOD	< LOD	0.03	0.09	0.38
2‐(N‐methyl‐perfluorooctane sulfonamido) acetic acid	MeFOSAA	All CAPS	260	47.3 (43.1, 51.5)			< LOD	< LOD	< LOD	0.04	3.77
		CAPS < 50 years	159	44.2 (39.0, 49.3)			< LOD	< LOD	< LOD	0.03	3.77
		Males < 50 years	131	42.8 (37.2, 48.4)			< LOD	< LOD	< LOD	0.03	3.77
		Females < 50 years	28	51.9 (38.1, 65.6)			< LOD	< LOD	0.01	0.04	0.54
Perfluoroheptanoic acid	PFHpA	All CAPS	193	35.1 (31.1, 39.1)			< LOD	< LOD	< LOD	0.04	0.71
		CAPS < 50 years	116	32.2 (27.4, 37.1)			< LOD	< LOD	< LOD	0.03	0.71
		Males < 50 years	98	32.0 (26.8, 37.3)			< LOD	< LOD	< LOD	0.03	0.71
		Females < 50 years	18	33.3 (20.3, 46.3)			< LOD	< LOD	< LOD	0.04	0.21
Perfluorooctane sulfonamide	FOSA	All CAPS	127	23.1 (19.6, 26.6)			< LOD	< LOD	< LOD	< LOD	0.19
		CAPS < 50 years	61	16.9 (13.1, 20.8)			< LOD	< LOD	< LOD	< LOD	0.19
		Males < 50 years	50	16.3 (12.2, 20.5)			< LOD	< LOD	< LOD	< LOD	0.19
		Females < 50 years	11	20.4 (9.3, 31.5)			< LOD	< LOD	< LOD	< LOD	0.03
Perfluorobutane sulfonic acid	PFBS	All CAPS	67	12.2 (9.4, 14.9)			< LOD	< LOD	< LOD	< LOD	1.78
		CAPS < 50 years	53	14.7 (11.0, 18.4)			< LOD	< LOD	< LOD	< LOD	1.78
		Males < 50 years	46	15.0 (11.0, 19.1)			< LOD	< LOD	< LOD	< LOD	1.78
		Females < 50 years	< 10	< 20			< LOD	< LOD	< LOD	< LOD	0.07
Perfluorododecanoic acid	PFDoDA	All CAPS	62	11.3 (8.6, 13.9)			< LOD	< LOD	< LOD	< LOD	0.21
		CAPS < 50 years	40	11.1 (7.8, 14.4)			< LOD	< LOD	< LOD	< LOD	0.21
		Males < 50 years	31	10.1 (6.7, 13.5)			< LOD	< LOD	< LOD	< LOD	0.16
		Females < 50 years	< 10	< 20			< LOD	< LOD	< LOD	< LOD	0.21
2‐(N‐ethyl‐perfluorooctane sulfonamido) acetic acid	EtFOSAA	All CAPS	29	5.3 (3.4, 7.1)			< LOD	< LOD	< LOD	< LOD	0.19
		CAPS < 50 years	10	2.8 (1.1, 4.5)			< LOD	< LOD	< LOD	< LOD	0.18
		Males < 50 years	< 10	< 10			< LOD	< LOD	< LOD	< LOD	0.18
		Females < 50 years	< 10	< 20			< LOD	< LOD	< LOD	< LOD	0.14

^a^ CAPS Limits of Detection (LODs) (ng/mL) were as follows: PFOA 0.014, PFOS 0.014, PFNA 0.008, PFHxS 0.019, PFDA 0.019, PFUnDA 0.006, MeFOSAA 0.005, PFHpA, 0.011, FOSA 0.004, PFBS 0.009, PFDoDA 0.013, EtFOSAA 0.006. Detection frequency (DF) is calculated as the proportion of samples above the LOD for each compound.

^b^ The first *p*‐value displayed for each PFAS tests the difference in geometric means between those under 50 versus 50 and older. The second *p*‐value displayed is for males versus females under age 50. Both contrasts were calculated using a *t*‐test on ln‐transformed PFAS values.

There was notable geographic variation in average serum concentrations and DFs among the CAPS state and sub‐state regions (Table S4). PFAS GMs tended to be higher in East‐coast regions than those from the Midwest and West (KS, MO and WA). North and Central NJ had higher PFOA GMs than other regions. North NJ had higher a PFHxS GM than other regions, and KS and MO had lower PFOA, PFOS, and PFNA GMs than all regions except WA. DFs varied much more for low‐detect compounds than for PFOA, PFOS, PFHxS, and PFNA (all regions > 99% DF), and PFDA and PFUnDA varied moderately by region. CT and southern ME had higher DFs of several short chain PFAS compared to other regions (MeFOSAA, PFHpA, FOSA (CT only), and PFDoDA). WA had > 75% detection of PFBS, whereas other regions were < 25%.

The 6 PFAS detected in over 75% of CAPS participants were moderately correlated (Table S5). Pearson correlation coefficients for natural log (ln)‐transformed PFAS ranged from 0.30 (PFUnDA and PFHxS, *p* < 0.0001) to 0.77 (PFDA and PFUnDA, *p* < 0.0001).

**Figure 1:**
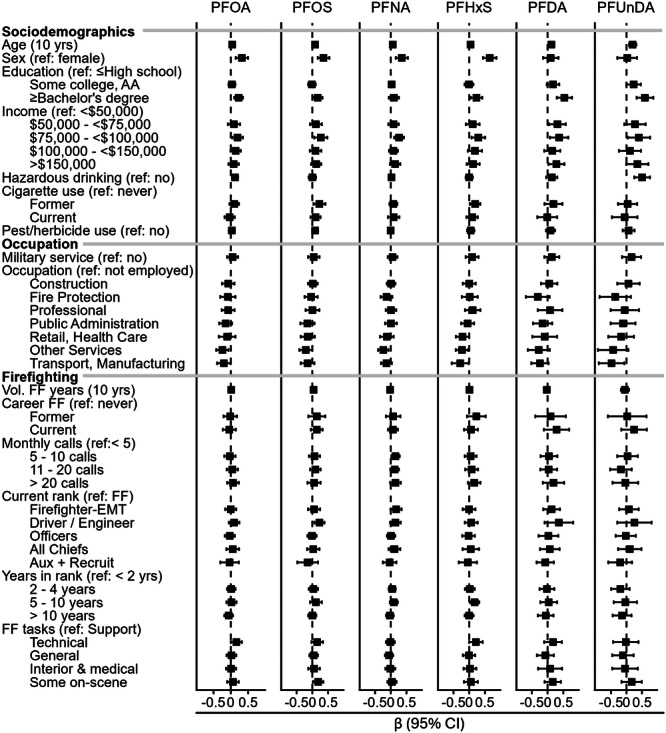
Adjusted associations between ln‐transformed serum PFAS and sociodemographic, occupational and fire service characteristics among CAPS volunteer firefighters (*n* = 550), estimated using generalized estimating equations. *Note:* Beta estimates and 95% confidence intervals (CIs) represent the change in ln‐transformed PFAS per change in the independent variable, adjusted for the other factors shown and accounting for clustering within fire departments. Aux, auxiliary; CAPS, Cancer Assessment and Prevention Study; EMT, emergency medical technician; FF, firefighter; PFAS, per‐ and polyfluoroalkyl substances.

**Figure 2:**
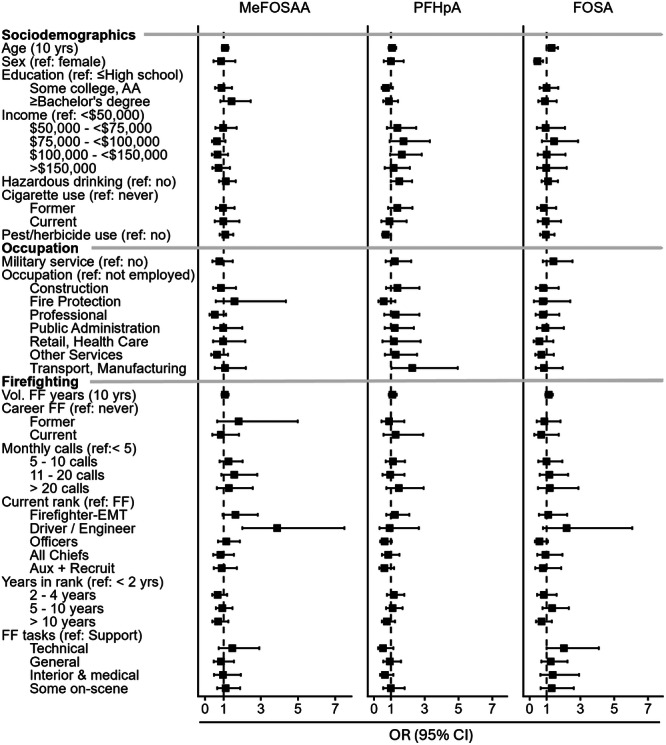
Adjusted associations between detected versus non‐detected serum PFAS and sociodemographic, occupational and fire service characteristics among CAPS volunteer firefighters (*n* = 550), estimated using generalized estimating equations. *Note:* Odds ratios (OR) and 95% confidence intervals (CIs) represent the odds of detecting the PFAS per change in the independent variable, adjusted for the other factors shown and accounting for clustering within fire departments. Aux, auxiliary; CAPS, Cancer Assessment and Prevention Study; EMT, emergency medical technician; FF, firefighter; PFAS, per‐ and polyfluoroalkyl substances.

We used multivariable GEE models to assess the associations between fire service, occupational, and sociodemographic factors with six ln‐transformed continuous PFAS and three dichotomous PFAS (detected vs. not detected), accounting for the correlation of individuals within fire departments (Figures 1 and 2 and Table S6).

We observed mostly null associations between PFAS and fire service variables, adjusting for occupational and sociodemographic factors in the GEE models (Figures 1 and 2). For most PFAS, there were no clear associations observed between years of volunteer fire service and serum PFAS. Compared to never career firefighters, there were some indications of positive associations between PFOS, PFDA, PFUnDA and current career firefighter status and with PFHxS and MeFOSAA with former career firefighter status. Monthly calls did not exhibit a dose‐response relationship with any PFAS. However, compared to those responding to fewer than five calls/month, those responding five or more calls had higher average serum PFNA, and responding to > 20 calls/month was positively associated with serum PFHxS. Driver operator/engineer (vs. firefighter) rank was positively associated with serum PFOS, PFNA, PFDA, PFUnDA, MeFOSAA, FOSA, though the latter three model estimates were imprecise. Compared to the command task class, the on‐scene and technical tasks class was positively associated with several PFAS, including PFOA, PFOS, PFHxS, PFDA, MeFOSAA, and FOSA. The some on‐scene tasks class had higher average PFOS compared with the command group. This class also showed slightly positive associations with PFDA and PFUnDA.

There were few associations between occupational characteristics and PFAS in adjusted GEE models (Figures 1 and 2). Transportation, wholesale trade and manufacturing industry workers tended to have lower PFAS levels, except for PFHpA, which had higher odds of detection in this group compared to the not employed. Other services workers tended to follow a similar pattern. Apart from MeFOSAA which was positively associated with fire protection, participants whose primary occupation was fire protection had lower or similar average PFAS levels to the not employed category.

There were several notable associations between sociodemographic and health factors in adjusted GEE models (Figures 1 and 2). Age was positively associated with all PFAS, except for MeFOSAA or PFHpA. Male sex was positively associated with PFOA, PFOS, PFNA, PFHxS, was negatively associated with FOSA, and associations with other compounds were near the null. Holding a bachelor's degree or higher was positively associated with all compounds except for PFHpA and FOSA, compared to having a high school education or below. Income appeared positively, but non‐linearly, associated with most compounds. Compared to those with an annual household income of < $50,000, those making $75,000–100,000 had higher average concentrations of the six continuous PFAS measures. We saw positive associations between serum PFOA, PFUnDA, PFHpA, and PFDA and hazardous drinking. Compared with non‐smokers, former cigarette smoking was positively associated with PFOS and PFHxS, and weakly with PFOA, PFNA, PFDA, and PFHpA. Past 12‐month pesticide/herbicide use was positively associated with PFOS, and negatively with PFHpA.

**Figure 3:**
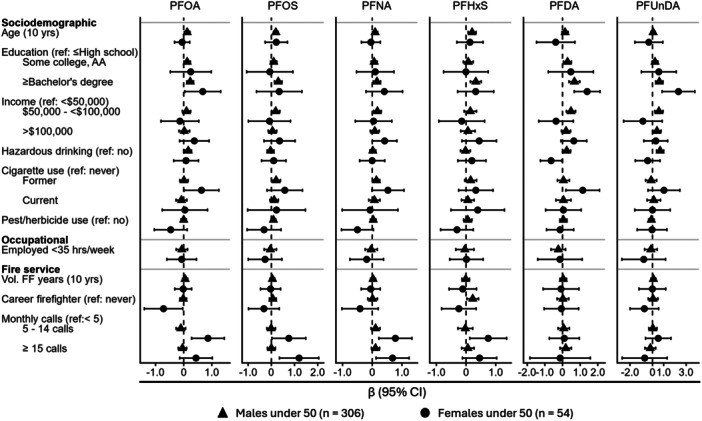
Adjusted associations between ln‐transformed serum PFAS and sociodemographic, occupational and fire service characteristics among CAPS volunteer firefighters under 50 years, stratified by sex, estimated using generalized estimating equations. *Note:* Beta estimates and 95% confidence intervals (CIs) represent the change in ln‐transformed PFAS per change in the independent variable, adjusted for the other factors shown and accounting for clustering within fire departments. CAPS, Cancer Assessment and Prevention Study; FF, firefighter; PFAS, per‐ and polyfluoroalkyl substances.

In sex‐stratified multivariable GEE models using a reduced covariate set, several patterns were consistent between males and females under age 50 as with the full cohort, though there were also notable differences (Figure 3 and Tables S7‐S8). For example, the highest level of education, and annual household income over $100,000 tended to be positively associated with most high‐detect PFAS, with stronger trends observed among females, but not males. Age was positively associated with increased levels of most PFAS for males but not females. Hazardous drinking behavior was positively associated with PFDA and PFUnDA among males and inversely associated with PFDA among females. Former smoker status was positively associated with several serum PFAS among both males and females with some stronger trends among females (PFNA, PFDA, PFUnDA), though confidence intervals were wider among females. Pesticide or herbicide use was weakly positively associated with PFOS among males, but inverse patterns of association were observed among females for PFOA, PFNA, with weaker inverse trends for PFOS and PFHxS. Similar to the full cohort, we did not observe associations between employment status and any of the high‐detect PFAS. Compared to those who had never been a career firefighter, current and former career firefighter status was positively associated with PFHxS among male volunteer firefighters, but inversely associated with PFOA among female volunteer firefighters, with inverse trends for PFOS, PFNA, PFHxS and PFUnDA also among females. Similar to the whole cohort, males under 50 showed positive associations between average monthly calls and PFNA. Females under 50 serum PFOA, PFOS, PFNA, and PFHxS were positively, though in some cases nonlinearly, associated with average monthly calls.

## 4 Discussion

This study of 550 US volunteer firefighters describes the profiles and predictors of this understudied occupational group's serum PFAS. There was expected and notable variation by sex, geographic region, and age in our study population's serum PFAS profiles. We also observed certain firefighting, occupational, sociodemographic, and health factors associated with CAPS volunteer firefighters' serum PFAS. Some of these predictors displayed different patterns of association among male and female volunteer firefighters.

### 4.1 PFAS Serum Profiles

Our cohort of volunteer firefighters generally had similar or lower average serum PFAS concentrations to those seen in studies of career firefighters and general populations without known point‐source contamination. Several studies of career firefighters have reported higher average concentrations of certain compounds than reference populations. However, there is heterogeneity in absolute and relative PFAS concentrations reported in these studies. For example, median serum PFOS has ranged from below 3.3 ng/mL in a 2019–2020 study of 110 Czech firefighters to 66.0 ng/mL among 149 AFF‐exposed Australian airport firefighters sampled in 2013 [18, 48]. These highest levels have generally been reported in firefighters with a significant history of AFFF exposure, such as airport firefighters. While we did not measure AFFF use, average PFAS concentrations of legacy PFAS among structural firefighters tend to be closer to general populations (e.g., those in NHANES). Burgess et al. (2023) [16] measured serum PFAS in 290 career firefighters from four US fire departments between 2016 and 2019. Both DFs and concentrations varied by department. Overall, Burgess et al. (2023) [16] reported elevated PFHxS, PFNA, sm‐PFOS, n‐PFOS, and n‐PFOA, with the largest differences observed for PFHxS, which was 2.5 times higher among male firefighters than that of an age‐ and sex‐matched NHANES referent (GMs: 3.78 vs. 1.48 ng/mL) and 3.3 times higher among female firefighters than the NHANES referent (GMs: 2.06 vs. 0.81 ng/mL). Among CAPS volunteer firefighters, we observed lower average PFHxS concentrations than those described by Burgess et al. (2023), though there are notable temporal differences between the populations in that study and CAPS, thus the differences may reflect population‐level declines that have been reported [49]. In addition, we similarly observed variation by geography and sex, as well as age. Due to age‐dependent and sex‐specific PFAS elimination pathways among pre‐menopausal females, including menstruation, lactation, and parity, our finding that females under age 50 had lower average serum PFAS concentrations compared to males under 50 was expected and consistent with existing literature [7, 16, 36, 39, 47, 50]. Beaucham et al. (2025) [51] studied serum PFAS levels in Maui County, Hawaii employees who responded to the 2023 wildfires. They observed elevated average PFHxS serum concentrations among career firefighters (median 1.2 ng/mL) compared to other occupations. These firefighters' median PFHxS concentration was similar to CAPS (median PFHxS 1.23 ng/mL). Dobraca et al. (2015) [52] studied 101 California career firefighters in 2010 and found that these firefighters had an average PFDA concentration three times higher than 2009–2010 NHANES males 20 years and older (0.9 vs. 0.3 ng/mL), however other PFAS compounds were lower or showed small differences between these firefighters and 2009–2010 NHANES adult males. We observed a GM PFDA concentration among CAPS of 0.07 ng/mL, whereas more recent cycles of NHANES (through 2017‐March 2020) show PFDA GMs for the US population between 0.15 and 0.20 ng/mL [49, 53]. The PFAS concentrations at which adverse health outcomes may occur are not well understood, and sensitive groups may experience adverse outcomes with a sum of PFOA, PFOS, PFNA, PFHxS, PFDA, PFUnDA, and MeFOSAA of 2.0 ng/mL and higher [54].

### 4.2 Associations Between Serum PFAS and Predictors of Interest

The ubiquity of PFAS often makes it difficult to pinpoint exposure sources. Nevertheless, we expected that not only fire service factors, but also other occupational and sociodemographic factors would be important predictors of serum PFAS in our study population. For example, in a pilot study of North Carolina career firefighters, PFAS exposures measured using silicone tags were mostly similar or lower on‐duty and off‐duty, with the exception of PFOS, which was 2.5 times higher while on‐duty and while responding to fires than measures collected off‐duty [55]. Two biomonitoring studies that examined predictors of serum PFAS in multivariable models found that the models explained a maximum of 32% of the variation in the firefighters' serum PFAS concentrations [48, 52]. Such findings likely indicate the importance of multiple exposure pathways.

### 4.3 Fire Service Factors

In the study of Maui County first responders, in addition to higher average PFHxS concentrations than other occupations, firefighters with longer tenures (≥ 30 years) had higher median concentrations of the sum of seven PFAS than those with shorter tenures. We did not observe significant associations between firefighting tenure (years of volunteer service) and serum PFAS in our multivariable models. This may be in part explained by the strong correlation between age and firefighting tenure and point to the importance of other factors in our population's PFAS exposure experience. Dobraca et al. (2015) [52] found PFHpA concentrations were positively associated with monthly or greater commercial fire responses, and that PFNA concentrations were associated with any (vs. no) hazardous materials response. We observed positive, nonsignificant associations between several compounds, including PFHpA, and > 20 monthly calls, and positive associations between PFNA and > 5 monthly calls (vs. < 5 calls). While we did not observe associations between task classes and PFNA, other PFAS (PFOA, PFOS, PFHxS, PFDA, MeFOSAA, and FOSA, though not PFHpA) were associated with the technical task class, of which hazmat was one component performed by a majority of class members.

Firefighting tasks with increased smoke exposure have been positively associated with PFAS concentrations in studies of career firefighters [12, 14, 15]. In a 2008 study of 458 WTC responders by Tao et al., PFOA and PFHxS plasma concentrations were higher in smoke‐exposed versus dust‐exposed responder groups (*p* ≤ 0.05), and that PFNA was higher among smoke‐exposed responders who were more smoke‐exposed versus less smoke‐exposed (*p* = 0.001) [14]. Among CAPS participants, we did not observe associations between participation in WTC response and serum PFAS, though this event occurred 20 years prior to our study. Associations between monthly calls and technical task class and some serum PFAS may offer support to the importance of smoke‐ or combustion‐related PFAS exposure pathways. In fact, nearly all members in the technical task class perform overhaul, ventilation, rapid intervention, fire attack, suppression, interior, and most also perform driver and pump panel operations.

Trowbridge et al. (2020) [15] found that among female firefighters in San Francisco, firefighter and officer rank were associated with higher PFNA, PFOA, PFDA, and PFUnDA concentrations compared with drivers. Interestingly, we observed positive associations between PFNA and driver operator/engineer rank (vs. firefighter), and officer rank did not differ from firefighters for these compounds. Anecdotally, drivers tend not to wear respiratory protection and may experience smoke exposure as a result. The lack of an observed association between serum PFAS and officer rank may be due to turnover in officer positions in the volunteer fire service. We did not have sufficient participants in several of these categories to include tasks or rank in sex‐stratified models.

We observed mostly null associations between career firefighter status and serum PFAS in the full cohort and among males under 50. Unexpectedly, we observed an inverse association between serum PFOA and being a former or current career firefighter among females under 50, with nonsignificant inverse associations with PFOS and PFNA as well.

While we did not measure AFFF use in CAPS, other studies of firefighters have reported AFFF use was positively associated with serum PFOA, PFOS, PFHxS, and PFNA [12, 15, 17, 18, 56]. In the United States, PFOS and PFOA were phased out of AFFF beginning in 2003 and 2010, respectively [21]. Various novel or replacement PFAS with less‐understood properties and health effects have been identified in newer fluorinated foam formulations [57], and legacy PFAS may still be detected in modern fluorotelomer firefighting foams, potentially as a manufacturing byproduct [21]. Many volunteer fire departments may have occasionally used or trained with AFFF and may retain older formulations. A 2024 survey of Maine fire departments in which 40% of respondents represented all volunteer departments found that nearly 70% had used or trained with AFFF prior to its state‐level ban in 2022, and 28% of departments had stored AFFF stock on site [58]. Just 13% of those surveyed departments reported acquiring fluorine‐free foams. More research is needed to understand these practices in other states. Furthermore, novel PFAS have been detected in firefighters' turnout gear, including 6:2FTS and other compounds [59, 60, 61]. The extent to which firefighters' exposure to PFAS through gear may occur is an area of ongoing investigation. In future studies, measuring PFAS historically and currently present in AFFF and replacement foams, and additional information about firefighting practices and activities which were not asked in our enrollment survey such as AFFF use, types of fire responses including a history of airport firefighting, PPE/gear use, laundering, and decontamination practices would be useful to assess among volunteer firefighters.

### 4.4 Occupational Factors

A growing number of studies have characterized occupational exposure to PFAS. A 2023 review found that the majority of occupational PFAS exposure studies were of fluorochemical workers and first responders, with a smaller number of studies of office settings, retail spaces, ski wax technicians, fishing industry workers, manufacturing, and others [9]. Mitchell et al. (2025) [62] reported higher average PFHxS, PFOS, and PFHpS concentrations among Arizona firefighters compared to other essential worker groups in the state, adjusted for age, sex, race, ethnicity, year of sample collection, and county. Similarly, Beaucham et al. (2025) [51] identified higher serum PFHxS among firefighters than other occupational groups in Maui, Hawaii. We assessed associations between current occupation categories and serum PFAS. Unexpectedly, compared to the reference category of not employed, few occupation categories showed positive associations with serum PFAS. We expected that both career firefighter status and Fire Protection occupation would be positively associated with serum PFAS. There were some positive trends for a few compounds, but CIs all included the null and there were inconsistent patterns between current and former career firefighters. In terms of Fire Protection occupation category, compared to the not employed, most estimates were negative or near the null.

Our occupation categories had low granularity. It is also possible that participants interpreted and answered the question in different ways. For example, Fire Protection was not a provided occupation category. There were 64 participants who identified as a current career firefighter in a separate question about career firefighter status, making it likely that some people whose primary occupation was firefighting selected ‘government’ or some other provided category, while some selected “other” and wrote in fire service‐related jobs. Similar phenomena may have occurred with other occupation categories, potentially leading to misclassification.

Employment may be linked to age or a healthy worker effect. Those of retirement age (65 and older) made up over 40% of the not employed group. Even though our models adjusted for age, retired participants may have had a longer history of occupational PFAS exposure than those who are still working. In addition, a relatively larger proportion of those over 65 (vs. under 65) in our population were former career firefighters, and we did observe positive associations between former career firefighter status and PFOS, PFHxS, and MeFOSAA. Anecdotally, many retirees, including former career firefighters, may join their local volunteer fire department. Overall, further investigation of occupational exposure to PFAS is an important direction for future work.

We did not observe associations between serum PFAS and a history of active‐duty military service. However, due to the length of time since most participants reported service and the short duration of most service, this is likely not the most appropriate population in which to assess associations between military service and serum PFAS.

### 4.5 Sociodemographic and Health Factors

Age was positively associated with nearly all compounds we examined among the whole cohort, though these associations were attenuated in males and females under age 50. Age has been consistently positively associated with long‐chain PFAS concentrations in studies of adult populations, possibly due to the longer half‐lives of these compounds and ongoing exposure [7, 63, 64].

We observed strong positive associations between male sex and most compounds. Females have been found to have lower average concentrations of most long‐chain PFAS compared to males, likely due to additional elimination pathways such as menstruation, childbirth and breastfeeding [7, 63, 64, 65, 66, 67, 68]. These elimination pathways may in part explain why CAPS female volunteer firefighters under age 50 had lower average concentrations of PFOA, PFOS, PFNA, and PFHxS than males.

Consistent with other studies, we saw that education and household income were positively associated with serum concentrations of PFOA and PFOS [69], in addition to other compounds in sex‐specific models. One hypothesis is that consumer purchasing at least partially drives this variation—for example, higher income households may more frequently purchase carpeting and furniture with stain‐resistant treatments or use outdoor gear that contain PFAS. Inconsistencies and nonlinear associations could reflect the changing landscape of PFAS in consumer products and consumer awareness or misclassification of household income.

Phan et al. (2022) [70] reported that from 1992 to 2019, the prevalence of cigarette smoking declined among firefighters and was lower in firefighters than other occupational groups, whereas SLT use was more prevalent among firefighters and law enforcement than in other occupational groups and the general population. Volunteer firefighters reportedly have a higher prevalence of cigarette smoking than career firefighters, but lower than the general population [71]. Barton et al. (2020) [64] reported an association between smoking history and serum PFAS concentrations that the authors hypothesized may be indicative of other occupational or behavioral PFAS exposure pathways rather than a direct exposure source. The positive trends we observed between former cigarette smoking and some PFAS may be consistent with that hypothesis since to our knowledge the presence of PFAS in tobacco products has not been reported.

Alcohol consumption may affect how PFAS are absorbed and eliminated [72, 73]. In addition to metabolic pathways, an observed relationship between alcohol consumption and serum PFAS may be a function of the volume of liquid consumed. One recent study detected PFAS in most of the domestic and international beers tested and identified a strong relationship between the amount of PFAS measured and whether the beers were brewed in areas with PFAS in drinking water supplies [74]. Over a third of our population of volunteer firefighters reported hazardous drinking behaviors. Other studies have also reported high prevalences of heavy and binge drinking, hazardous drinking, or alcohol use disorder among firefighters compared to non‐firefighter populations [27, 75, 76, 77]. We observed positive associations between serum PFOA, PFUnDA, PFHpA, and PFDA and hazardous drinking behavior. PFOA and PFHxS were positively associated with alcohol consumption in one study using NHANES data [73]. The relationships between types and amount of alcohol consumption, PFAS exposure, and health outcomes warrants further investigation, and may partially explain why we observed different relationships among female volunteer firefighters compared to males.

Past 12‐month pesticide/herbicide use was positively associated with PFOS in our study, both overall and among male firefighters under 50. PFAS have been used in pesticide, herbicide, and fertilizer applications as both active and inactive (i.e., surfactant) ingredients [4, 78]. PFAS have also been detected in high‐density polyethylene (HDPE) containers used for storage of pesticides and other products and were shown to leach into stored liquids [79]. PFOS and PFOA were found to be higher among pregnant greenhouse workers than two general population comparison populations [80]. Interestingly, we observed inverse associations between pesticide/herbicide use and PFNA, and inverse though not statistically significant associations with PFOA among female firefighters under age 50. These differences may reflect different products used, use patterns, or different PPE or decontamination practices among female compared to male firefighters.

### 4.6 Strengths and Limitations

This is the first study of volunteer firefighters of this size that combines survey and biological data to evaluate occurrence and predictors of PFAS exposure. In addition to legacy compounds, we were able to assess serum profiles and predictors of compounds less frequently reported in the literature, including PFDA, PFUnDA, MeFOSAA, PFHpA, and FOSA. Furthermore, we had a relatively large number of female volunteer firefighters represented in our study and were able to conduct sex‐specific analyses. Our use of LCA leveraged detailed firefighting task data by identifying groups that may be targeted for future study or exposure reduction efforts. Volunteer firefighters are a unique occupational population as many volunteer firefighters have primary occupations other than firefighting. These approaches may be applied to other populations who may experience occupational exposures in their secondary occupations or volunteer work. Our results are broadly consistent with the existing firefighter, occupational, and general population biomonitoring literature, while also exploring novel aspects of the volunteer firefighter exposure experience.

This cross‐sectional study is subject to some limitations. First, we measured a panel of 12 PFAS analytes at one time point. Some evidence suggests that firefighters may be exposed to other PFAS not routinely included in targeted panels [60, 81]. There is also growing concern about exposure to novel or replacement PFAS and their metabolites (e.g., trifluoroacetic acid [TFA], among others) [82], and these exposures have not been widely quantified in firefighter biomonitoring studies. In addition, PFAS with shorter half‐lives may not have been detected, however, this does not mean an individual has not been exposed to a particular PFAS. Future work using nontargeted analyses is of interest, as are ongoing biomonitoring efforts. Some of the predictors assessed, particularly fire service factors, can change frequently and may not be indicative of the status when exposure to PFAS with long half‐lives may have occurred. In general, we did not have data which could enable a direct assessment of exposure pathways, such as dietary or drinking water measures [63, 67, 83, 84]. Some of the factors assessed are either not modifiable or are proxy measures for true exposure sources (e.g., age, socioeconomic status, monthly fire calls). We did not measure several firefighting‐related variables of interest such as AFFF use, PPE or turnout gear, decontamination practices, or types of call responses in our survey. Although CAPS participants appear similar to what is known about the volunteer fire service (e.g., firefighting tenure, age, sex) [24], selection bias may have occurred from the referral sampling approach. For example, if participants were more highly educated or healthier than non‐participants, average PFAS concentrations may have been over‐ or under‐estimated in our study. In addition, we did not adjust for multiple comparisons, as the objective of this exploratory analysis was to identify trends in an understudied population rather than to carry out hypothesis testing. As such, results should not be over‐interpreted. Finally, we selected a GEE model to estimate population‐level associations between predictors of interest and serum PFAS, however this may mask intra‐departmental variability. Future studies may choose to allow for variability using other model approaches (e.g., mixed effects) or otherwise investigate variation in regions or departments.

Important future research with volunteer firefighters may include collecting additional information about PFAS exposures and predictors of exposure not captured in this study (i.e., AFFF use), collecting data about non‐participation, and additional, ongoing biomonitoring using novel techniques to better understand volunteer firefighters' exposures.

### 4.7 Conclusions

This exploratory analysis assessed volunteer firefighters' serum PFAS profiles and identified several predictors of PFAS exposure among an understudied majority of the US fire service. We observed variation in volunteer firefighters' PFAS exposure profiles by sex, age, and geography, as well as associations between serum PFAS and sociodemographic, health, occupational, and fire service characteristics. These findings highlight the likely multifaceted exposure experience of these first responders and possibly different pathways of exposure as well as elimination between male and female volunteer firefighters.

Exposure reduction and mitigation efforts directed at the fire service and the general population are both likely important in reducing volunteer firefighters' PFAS exposures. In addition, variation in PFAS exposure profiles not only underscores the importance of tailored exposure reduction efforts, but also the need to understand how these exposures may affect volunteer firefighters' disease risk.

## Author Contributions

Katherine A. Lubina, Zorimar Rivera‐Núñez, Miriam M. Calkins, Robert Laumbach, Shou‐En Lu, Jefferey L. Burgess, Jaclyn M. Goodrich, Casey C. Grant, and Judith M. Graber contributed to the conceptualization or design of the work, including funding acquisition. Katherine A. Lubina, Shou‐En Lu, Elena Austin, Derrick L. Edwards, Maria D. H. Koeppel, Zhihua (Tina) Fan, Chang Ho Yu, Kaleigh M. Hinton, Brian Kubiel, Daniel Roy, and Judith M. Graber contributed to the acquisition, analysis, or interpretation of data for the work. Katherine A. Lubina drafted the manuscript, and all other authors contributed to the critical revision of the work for important intellectual content. All authors gave final approval for the version to be published and agreed to be accountable for all aspects of the work.

## Disclosure

The findings and conclusions in this paper are those of the authors and do not necessarily represent the official position of the National Institute for Occupational Safety and Health (NIOSH), Centers for Disease Control and Prevention.

## Ethics Statement

Participants provided written informed consent at enrollment. Institutional Review Boards at the University of Miami, the University of Arizona, and Rutgers, the State University of New Jersey, approved this research project (STUDY00002192, Pro2019000811).

## Conflicts of Interest

The authors declare no conflicts of interest.

## Institution at Which the Work Was Performed

Rutgers, The State University of New Jersey.

## Supporting information

Supporting File

## Data Availability

The data that support the findings of this study are available on request (https://www.ffccs.org/contact). Data requests will be reviewed by the study's Oversight and Planning Board to address firefighter concerns prior to determination of sharing de‐identified data.
