# Supplementary material for: Per‐ and Polyfluoroalkyl Substances (PFAS) Exposure Profiles and Their Predictors in a Study of US Volunteer Firefighters
Source: Am J Ind Med. 2026 Jun 14;69(9):745–64. doi: 10.1002/ajim.70104 (PMC13456399; doi:10.1002/ajim.70104)
Supplement: Supplementary file 1 [file AJIM-69-745-s001.docx]

| **Supplemental Table 1. Variable use, management and categorization based on CAPS survey data.** | | | | | |
| --- | --- | --- | --- | --- | --- |
| **Domain** | **Variable of interest** | **Form used** | **Description** | **In final models** | **Original survey question (response options)** |
| Fire Service | Years of volunteer fire service | Continuous | Calculated as the difference between start date in volunteer fire service and survey date for active members | X | What year did you begin volunteering as a full member with any fire department? |
|  | Career firefighter status | Categorical | Former, current, or never a career firefighter | X | Have you ever worked as a career firefighter (for pay)? (1. No 2. Yes, I am currently a career firefighter 3. Yes, I am a former career firefighter |
|  | Current volunteer rank | Categorical | Rank re-categorized into the following based on distributions 1) Firefighter (includes: fire investigator, instructor) 2) Firefighter/EMT  3) Driver operator/engineer 4) Officers (includes: captain, lieutenant)  5) Chiefs (includes: Assistant, Battalion, and Fire Chiefs) 6) Recruits and auxiliary members | X | What is your current rank with [fire department]? (1. Firefighter 2. Firefighter/Paramedic/EMT 3. Driver Operator 4. Inspector 5. Fire Investigator 6. Lieutenant 7. Captain 8. Battalion Chief 9. Deputy Chief 10. Other Chief 11. Fire Chief 12. Paramedic/EMT Chief 13. Fire Police 14. Other (please specify)) |
|  | Years in current rank | Categorical | Categorized based on distribution:  0-1 years 2-4 years 5-10 years >10 years | X | How long have you been in your current rank? (years) |
|  | Average number of monthly calls | Categorical | Categorized based on distribution:  < 5 calls/month 5 – 10 calls/month 11 – 20 calls/month > 20 calls/month | X | On average, how many fire department calls do you respond to in a month? |
|  | Average number of hours spent responding to calls per month | Categorical | Categorized based on distribution:  0-5 hours/month 6-15 hours/month 16-30 hours/month >30 hours/month |  | On average, how many hours do you spend responding to fire department calls per month? Note: please include all time from when you receive a call until you leave the fire station after the call |
|  | Average number of hours spent training per month | Categorical | Categorized based on distribution:  <5 hours/month 5-8 hours/month 9-14 hours/month ≥15 hours/month |  | On average, how many hours do you spend in fire department training per month? |
|  | Average number of hours spent on other department tasks per month | Categorical | Categorized based on distribution:  0-5 hours/month 6-10 hours/month 11-25 hours/month >25 hours/month |  | On average, how many hours do you spend at the fire station for other reasons per month? (e.g., meetings, fundraisers, cleaning) |
|  | Tasks performed on fire calls classes | Categorical | Categorized into 5 latent classes: 1) Command, investigation, inspection, on-scene auxiliary support, recruit/probationary, off-scene support 2) On-scene and technical FF tasks 3) On-scene - general FF 4) Interior and medical 5) Some on-scene exterior and interior | X | What are your current job tasks on fire calls? (1. Interior 2. Driver 3. Engineer 4. Forcible entry 5. Pump panel 6. Medical calls/disease exposure 7. Vehicle extraction 8. Hazmat 9. Fire attack 10. Fire suppression 11. Rapid intervention 12. Search and rescue 13. High angle rescue 14. Overhaul 15. Trench rescue 16. Technical rescue response 17. Water rescue 18. Ventilation 19. Wildland brushfire 20. Other (please specify) ) |
| Employment | Employment status | Categorical | Calculated from average number of hours worked per week (for pay):  Full-time (≥35 hours/week) Part time (1 – 34 hours per week) Not employed (0 hours per week), or indicated not employed |  | Are you currently employed? (1. Yes 2. No) How many hours per week on average do you work? |
|  | Primary occupation category | Categorical | Current primary occupation type coded using the North American Industry Classification System (NAICS) 2022.^33^ Categories were created using the first two or three digits of the six-digit NAICS code to create groups of at least 50. Fire service (n = 36) was the one exception due to relevance of this occupation to the research question. Categories created using this NAICS framework were:  1) Construction, Agriculture, Forestry, Fishing, Mining, Services to Buildings 2) Fire Protection 3) Professional, Finance, Education, Information 4) Public Administration, Justice/Public Order 5) Retail, Health Care, Arts, Entertainment, Recreation, Accommodation 6) Other Services (e.g., service worker, auto mechanic) 7) Transportation, Wholesale Trade, Manufacturing 8) Not employed.   ‘Other’ occupations reported using text entry (n = 154) were classified by two coders independently using NAICS. Classifications were then compared. In the event of disagreement between the classifications (n = 49 responses), NAICS documentation for the selected categories was consulted in detail to identify which category best fit the job description provided. If a determination could not be made or two categories seemed to fit the equally well, a third reviewer was consulted (n = 8).  If participants selected multiple categories to describe their primary occupation (n = 38), the category that fit best based on their reported primary job tasks performed was assigned such that primary occupation categories would be mutually exclusive. | X | What type of business or what service do they provide? (1. Manufacturing 2. Clerical 3. Retailer 4. Service provider 5. Construction 6. Mining 7. Farming/Fishing 8. Government 9. Landscaping 10. Mechanic 11. Restorer 12. Roofing 13. Painter 14. Plumbing 15. Other (please specify)) |
|  | More than 1 job | Dichotomous | Participants who reported working a second or third job were categorized as having more than 1 job. |  | Do you currently have a second job? (1. Yes 2. No) |
|  | Active duty military service | Dichotomous | Yes: any history of active duty military service beyond basic training; No: no history of active duty military service beyond basic training. | X | Have you ever served on active duty in the U.S. Armed Forcesor other uniformed services? Active duty includes serving in the U.S. Armed Forces as well as activation from the reserves, National Guard, or Coast Guard. (1. Yes, on active duty in the past, but not now 2. Yes, now on active duty 3. No, never on active duty expect for initial/basic training 4. No, never served in the U.S. Armed Forces) |
|  | Time since active duty military service | Categorical | Calculated as the difference of the end of active-duty service and the survey date and categorized based on the distribution: 0-15 years, 16 - 35 years, >35 years |  | In what year were you last released from active duty? |
|  | Total years of active duty | Categorical | Calculated as the difference of the start and end of active-duty service and categorized based on the distribution as < 1 year vs. > 1 year |  | In what year did you first enter active duty? |
|  | Participation in 9/11/01 World Trace Center (WTC) rescue and recovery efforts | Dichotomous | Present or not at WTC response operations. |  | Were you present during fire ground operations (including fire suppression, search and rescue, crowd control, clean-up, etc.) at the World Trade Center following the terrorist attacks on 9/11/2001? (1. Yes 2. No) |
| Sociodemographic | Age in years | Continuous | Calculated using date of birth to survey date | X | (Calculated) |
|  | Sex | Dichotomous | Sex assigned at birth, male or female | X | What was your sex at birth? (1. Male 2. Female) |
|  | Race and ethnicity | Dichotomous | Race and ethnicity was grouped into non-Hispanic White and all other racial and ethnic identities (‘other’) due to a high proportion of non-Hispanic White participants. |  | Do you consider yourself Hispanic or Latino? (1. Yes, 2. No, 3. Not sure/don't know) Which one or more of the following would you say is your race? (1. White 2. Black or African American 3. Asian 4. Native Hawaiian or other Pacific Islander 5. American Indian or Alaska Native 6. Other (free response) 7. Not sure/don't know) |
|  | Education | Categorical | Highest level of education obtained (≤ high school diploma or equivalent; some college, associate degree, or trade school; and ≥ 4-year college degree) | X | What is the highest grade or year of school you completed? (1. Never attended school 2. Grades 1 to 8 (Elementary) 3. Grades 9 to 11 (Some high school) 4. Grade 12 or GED (High school graduate) 5. College 1 year to 3 years (Some college or technical school; Associates Degree) 6. College 4 years or more (College graduate) 7. Not sure/don't know) |
|  | Annual household income | Categorical | Categorized based on distribution: < $50,000, $50,000 - <$75,000, $75,000 - <$100,000, $100,000 - <$150,000, ≥$150,000 | X | What is your annual household income from all sources? (1. Less than $25,000 2. $25,000 - $35,000 3. $35,000 - $50,000 4. $50,000 - $75,000 5. $75,000 - $100,000 6. $100,000 - $150,000 7. $150,000 - $250,000 8. $250,000 - $500,000 9. > $500,000 10. Not sure/don't know) |
| Health and health behaviors | Body Mass Index (BMI) kg/m^2^ | Categorical | Calculated using self-reported height and weight, using the equation weight (lb) / [height (in)]^2^ x (703 (kg/m^2^)/(lb/in^2^)). ^34^ Standard BMI categories were used for modeling:  < 25.0 kg/m^2^  25-29.9 kg/m^2^ 30-34.5 kg/m^2^ ≥35.0 kg/m^2^ |  | About how much do you weigh without shoes? (Pounds) About how tall are you without shoes? (Feet) About how tall are you without shoes? (Inches) |
|  | Cigarette smoking | Categorical | Current cigarette smokers reported smoking at least 100 cigarettes in their lifetime and currently smoke cigarettes daily or some days.  Former smokers smoked at least 100 cigarettes in their lifetime and do not currently smoke cigarettes at all.  Never users denied ever smoking a cigarette or smoked 100 cigarettes or fewer in their lifetime. | X | In your entire life, have you ever smoked a cigarette, even just a puff ? (1. Yes 2. No 3. Not sure/don't know) In your entire life, have you smoked 100 or more cigarettes? (1. Yes 2. No 3. Not sure/don't know) Do you now smoke cigarettes? (1. No 2. Some days 3. Every day 4. Not sure/don't know) |
|  | Smokeless tobacco (SLT) use | Categorical | Never users of SLT had never tried SLT or had used SLT < 20 times.  Former users had used SLT >20 times but reported no use in the past 30 days.  Current users had used SLT >20 times and had used SLT in the past 30 days. |  | Have you ever used smokeless tobacco? (1. Yes 2. No 3. Not sure/don't know) Have you used smokeless tobacco at least 20 times in your entire life? (1. Yes 2. No 3. Not sure/don't know) During the past 30 days, on how many days did you usesmokeless tobacco? |
|  | Hazardous drinking | Dichotomous | Yes if Alcohol Use Disorders Identification Test-Concise (AUDIT-C) score of ≥3 for females and ≥4 for males ^35,36^ | X | How often do you have a drink containing alcohol? (0. Never 1. Monthly or less 2. 2-4 times a month 3. 2-3 times a week 4. 4 or more times a week) How many standard drinks containing alcohol do you have on a typical drinking day? Note: A standard drink is 12 ounces of beer, 5 ounces of wine,or 1.5 ounces of distilled spirits. A 40 ounce beer would countas 3 drinks, or a cocktail drink with 2 shots would count as 2 drinks. (0. 0 to 2, 1. 3 or 4, 2. 5 or 6, 3. 7 to 9, 4. 10 or more) How often do you have six or more drinks on one occasion? (1. Never 2. Less than monthly 2. Monthly 3. Weekly 4. Daily or almost daily) |
|  | Pesticide/herbicide use | Dichotomous | Yes if used pesticides or herbicides > 0 days in the past 12 months; No if used pesticides and herbicides 0 days in the past 12 months. | X | How many days in the past 12 months did you personally handle pesticides at your home? How many days in the past 12 months did you personally handle these herbicides? (e.g. weed killer, Round Up) |
| Other | Home is within 5 miles of military base or airport | Dichotomous | Yes or no. Don’t know responses were set to missing (n = 7). |  | Do you live near an airport or military airbase (i.e. within 5 miles)? (1. Yes 2. No 3. Not sure/don't know) |
|  | Region | Categorical | Created based on geographically proximate fire department locations for a minimum of 20 participants in a region. Used to calculate serum PFAS detection frequency and concentrations by region. |  | N/A |
|  | Fire department | Categorical | Fire department or company from which participant was recruited | X | What fire department are you affiliated with?  (If applicable) What company do you belong to? |
| **Notes**: Not sure/don't know responses were set to missing and imputed for all questions with not sure/don't know responses. 33. U.S. Census Bureau. North American Industry Classification System (NAICS), 2022 Edition. https://www.census.gov/naics/ 34. Centers for Disease Control and Prevention. About Adult BMI. U.S. Department of Health & Human Services. Updated June 3, 2022. https://www.cdc.gov/healthyweight/assessing/bmi/adult_bmi/index.html 35. Bush K, Kivlahan DR, McDonell MB, Fihn SD, Bradley KA. The AUDIT alcohol consumption questions (AUDIT-C): an effective brief screening test for problem drinking. Ambulatory Care Quality Improvement Project (ACQUIP). Alcohol Use Disorders Identification Test. Arch Intern Med. Sep 14 1998;158(16):1789-95. doi:10.1001/archinte.158.16.1789 36. O'Connor EA, Perdue LA, Senger CA, et al. Screening and Behavioral Counseling Interventions to Reduce Unhealthy Alcohol Use in Adolescents and Adults: Updated Evidence Report and Systematic Review for the US Preventive Services Task Force. Jama. Nov 13 2018;320(18):1910-1928. doi:10.1001/jama.2018.12086 | | | | | |

**Supplemental Figure 1**. Firefighter Cancer Assessment and Prevention Study (CAPS) participant selection diagram for present analysis.

| **Supplemental Table 2.** Sociodemographic, health, occupational, and firefighting characteristics of Firefighter Cancer Assessment and Prevention Study (CAPS) participants under age 50, stratified by sex | | | | |
| --- | --- | --- | --- | --- |
|  | **Males < 50 (n = 306)** | | **Females < 50 (n = 54)** | |
|  | **n** | **% (95% CI)** | **n** | **% (95% CI)** |
| **Demographic and health characteristics** |  |  |  |  |
| Age (years, mean (95% CI)) | 306 | 33.5 (32.5, 34.4) | 54 | 31.8 (29.5, 34.1) |
| Non-Hispanic white | 266 | 86.9 (83.1, 90.7) | 47 | 87.0 (77.8, 96.3) |
| BMI^1^ |  |  |  |  |
| Healthy and underweight (<25.0 kg/m2) | 55 | 18.0 (13.6, 22.3) | 22 | 40.7 (27.2, 54.3) |
| Overweight (25.0-29.9 kg/m2) | 119 | 38.9 (33.4, 44.4) | 17 | 31.5 (18.7, 44.3) |
| Obesity (≥ 30 kg/m2) | 132 | 43.1 (37.6, 48.7) | 15 | 27.8 (15.4, 40.1) |
| Education |  |  |  |  |
| High school or below | 83 | 27.2 (22.2, 32.2) | <10 | < 15 |
| Some college, associates, technical | 132 | 43.3 (37.7, 48.9) | 26 | 48.1 (34.4, 61.9) |
| Bachelor's degree or above | 90 | 29.5 (24.4, 34.7) | >20 | > 35 |
| Annual household income |  |  |  |  |
| < $50,000 | 69 | 24.0 (19.1, 29.0) | 20 | 39.2 (25.3, 53.1) |
| $50,000 - <$100,000 | 105 | 36.6 (31.0, 42.2) | 16 | 31.4 (18.2, 44.6) |
| >$100,000 | 113 | 39.4 (33.7, 45.1) | 15 | 29.4 (16.5, 42.4) |
| WTC Present^2^ | < 10 | < 5.0 | 0 | 0.0 (0.0, 0.0) |
| Pesticide/herbicide use in past year | 130 | 45.1 (39.4, 50.9) | 15 | 29.4 (16.5, 42.4) |
| Cigarette use |  |  |  |  |
| Never | 232 | 77.6 (72.8, 82.3) | >30 | > 65 |
| Former | 34 | 11.4 (7.8, 15.0) | <10 | < 20 |
| Current | 33 | 11.0 (7.5, 14.6) | <10 | < 15 |
| Smokeless tobacco use |  |  |  |  |
| Never | 194 | 70.5 (65.1, 76.0) | >40 | > 80 |
| Former | 38 | 13.8 (9.7, 17.9) | <10 | < 10 |
| Current | 43 | 15.6 (11.3, 20.0) | <10 | < 10 |
| Hazardous drinking^3^ | 123 | 40.2 (34.7, 45.7) | 18 | 33.3 (20.3, 46.3) |
| Home <5 miles of military base/airport | 58 | 19.4 (14.9, 23.9) | <10 | < 15 |
| **Occupational characteristics** |  |  |  |  |
| Military service^4^ | 29 | 9.5 (6.2, 12.8) | 0 | 0.0 (0.0, 0.0) |
| Employed full time (≥ 35 hours/week) | 254 | 87.0 (83.1, 90.9) | 37 | 71.2 (58.4, 83.9) |
| More than 1 job | 63 | 21.6 (16.9, 26.4) | 10 | 19.6 (8.3, 30.9) |
| Current occupation categories^5^ |  |  |  |  |
| Construction/Agriculture/Mining/Building Svcs | 56 | 19.4 (14.8, 24.0) | <10 | < 10 |
| Fire Protection | 29 | 10.0 (6.5, 13.5) | <10 | < 10 |
| Professional, Finance, Education, Information | 35 | 12.1 (8.3, 15.9) | 11 | 21.6 (9.9, 33.3) |
| Public Administration, Justice / Public Order | 50 | 17.3 (12.9, 21.7) | <10 | < 10 |
| Retail, Health Care, Arts/Hospitality, Other Svcs | 70 | 24.2 (19.3, 29.2) | 22 | 43.1 (29.1, 57.2) |
| Transportation, Wholesale, Manufacturing | 34 | 11.8 (8.0, 15.5) | <10 | < 10 |
| Not employed | 15 | 5.2 (2.6, 7.8) | <10 | < 20 |
| **Firefighting (FF) characteristics** |  |  |  |  |
| Volunteer FF experience (years, mean (95% CI)) | 304 | 10.5 (9.5, 11.6) | 54 | 6.9 (4.9, 9.0) |
| Current volunteer rank ^6^ |  |  |  |  |
| Firefighter | 166 | 54.4 (48.8, 60.0) | 22 | 40.7 (27.2, 54.3) |
| Firefighter / EMT | 48 | 15.7 (11.6, 19.8) | 22 | 40.7 (27.2, 54.3) |
| Officers | 53 | 17.4 (13.1, 21.7) | <10 | < 15 |
| All Chiefs | 26 | 8.5 (5.4, 11.7) | 0 | 0.0 (0.0, 0.0) |
| Auxiliary + Recruit | 12 | 3.9 (1.7, 6.1) | <10 | < 15 |
| Years in current volunteer FF rank |  |  |  |  |
| 0 - 1 years | 102 | 33.4 (28.1, 38.8) | 23 | 42.6 (29.0, 56.2) |
| 2 - 4 years | 113 | 37.0 (31.6, 42.5) | 16 | 29.6 (17.0, 42.2) |
| ≥ 5 years | 90 | 29.5 (24.4, 34.7) | 15 | 27.8 (15.4, 40.1) |
| Average monthly FF calls |  |  |  |  |
| < 5 calls | 64 | 22.8 (17.8, 27.7) | 21 | 40.4 (26.6, 54.2) |
| 5 - 14 calls | 99 | 35.2 (29.6, 40.9) | 12 | 23.1 (11.2, 34.9) |
| ≥ 15 calls | 118 | 42.0 (36.2, 47.8) | 19 | 36.5 (23.0, 50.1) |
| Ever a career firefighter (current or former)^7^ | 49 | 16.1 (11.9, 20.2) | <10 | <20 |
| FF task classes ^8^ |  |  |  |  |
| Support | 52 | 17.6 (13.3, 22.0) | 24 | 45.3 (31.4, 59.1) |
| Technical | 40 | 13.6 (9.6, 17.5) | <10 | <15 |
| General | 93 | 31.5 (26.2, 36.9) | 14 | 26.4 (14.1, 38.7) |
| Interior, medical | 54 | 18.3 (13.9, 22.7) | <10 | <15 |
| Some on-scene | 56 | 19.0 (14.5, 23.5) | <10 | <15 |
| State/regional group (# of depts.) |  |  |  |  |
| CT, MD, NJ (17) | 152.0 | 49.7 (44.0, 55.3) | 16.0 | 29.6 (17.0, 42.2) |
| ME (17) | 39.0 | 12.7 (9.0, 16.5) | 12.0 | 22.2 (10.8, 33.7) |
| KS + MO + IL (7) | 30.0 | 9.8 (6.5, 13.2) | 10.0 | 18.5 (7.8, 29.2) |
| TN (16) | 47.0 | 15.4 (11.3, 19.4) | < 10 | < 20 |
| WA (5) | 38.0 | 12.4 (8.7, 16.1) | < 10 | < 20 |
| Notes: Cells with fewer than 10 participants were suppressed for privacy reasons. 1. BMI: Body mass index, calculated using the equation weight (lb) / [height (in)]2 x (703 (kg/m2)/(lb/in2)). ^34^ 2. WTC: World Trade Center rescue and recovery efforts following 9/11/2001. 3. Hazardous drinking based on AUDITC score (≥3 for females and ≥4 for males) ^35, 36^ 4. Military service includes current or former active duty in U.S. military, beyond basic training. 5. Current primary occupation categories were created from survey responses using the North American Industry Classification System (NAICS).^33^ Ag: Agriculture contains forestry and fishing industry occupations. Hospitality includes entertainment, accommodation, and recreation industry occupations. 6. Firefighter rank includes investigators, instructors, and driver/operator engineers due to small numbers of individuals in those categories (<10). 7. All participants were current volunteer firefighters. Some were concurrently career firefighters in addition to their volunteer duties, and some were former career firefighters. 8. FF task classes created using latent class analysis. | | | | |

| **Supplemental Table 3. Model fit assessments for latent class analysis groupings of CAPS participants firefighting tasks categories (n = 536)** | | | | | | | | | |
| --- | --- | --- | --- | --- | --- | --- | --- | --- | --- |
| Model (# of classes) | Posterior probability (of inclusion in each class among those in the class) | Max. log likelihood | AIC | BIC | Entropy | Relative Entropy | Smallest Class % | LMR p-value | LMR Meaning |
| 1 | -- | -6491.5 | 13037.1 | -- | 0.00 | -- | -- | -- | -- |
| 2 | Class 1: 0.99; Class 2: 0.99 | -4606.2 | 9322.5 | 9558.1 | 11.05 | 0.97 | 34.7 | < 0.001 | 2 > 1 |
| 3 | Class 1: 0.95; Class 2: 0.99; Class 3: 0.96 | -4282.1 | 8730.2 | 9085.8 | 47.08 | 0.92 | 30.6 | < 0.001 | 3 > 2 |
| 4 | Class 1: 0.99; Class 2: 0.98; Class 3: 0.97; Class 4: 0.93 | -4168.5 | 8559.1 | 9034.6 | 46.58 | 0.94 | 11.7 | < 0.001 | 4 > 3 |
| 5 | Class 1: 0.98; Class 2: 0.96; Class 3: 0.95; Class 4: 0.94; Class 5: 0.91 | -4064.2 | 8406.3 | 9001.8 | 66.84 | 0.92 | 11.6 | < 0.001 | 5 > 4 |
| 6 | Class 1: 0.98; Class 2: 0.93; Class 3: 0.93; Class 4: 0.95; Class 5: 0.96; Class 6: 0.95 | -3989.1 | 8312.3 | 9027.7 | 73.76 | 0.92 | 5.6 | < 0.001 | 6 > 5 |
| Notes: LMR: Lo-Mendell-Rubin ad-hoc adjusted likelihood ratio test compares the current model to one with k-1 profiles | | | | | | | | | |

**Supplemental Figure 2**. Probability of latent class membership for each firefighting task.

| **Supplemental Table 4**. Detection frequencies (DFs), geometric means (GMs), and 95% confidence intervals (95%CIs) of serum PFAS measures among CAPS (n = 550), stratified by region | | | | | | | | | |
| --- | --- | --- | --- | --- | --- | --- | --- | --- | --- |
| PFAS | Region | n >LOD | DF % (95% CI) | GM (95% CI) | min | Q1 | Q2 | Q3 | max |
| PFOA | All (550) | 549 | 99.8 (99.5, 100.0) | 1.28 (1.21, 1.36) | <LOD | 0.83 | 1.32 | 1.96 | 9.25 |
|  | Maine - Central | 39 | 100.0 (100.0, 100.0) | 1.27 (1.09, 1.49) | 0.29 | 1.02 | 1.34 | 1.84 | 2.99 |
|  | Maine - South | 45 | 100.0 (100.0, 100.0) | 1.25 (1.08, 1.45) | 0.31 | 0.98 | 1.29 | 1.60 | 9.25 |
|  | Connecticut | 29 | 100.0 (100.0, 100.0) | 1.31 (1.10, 1.55) | 0.40 | 1.04 | 1.30 | 1.72 | 3.62 |
|  | New Jersey - Central | 50 | 100.0 (100.0, 100.0) | 2.27 (2.00, 2.58) | 0.75 | 1.76 | 2.20 | 3.01 | 7.34 |
|  | New Jersey - Coastal | 110 | 100.0 (100.0, 100.0) | 1.45 (1.31, 1.60) | 0.13 | 1.04 | 1.50 | 2.05 | 5.26 |
|  | New Jersey - North | 60 | 100.0 (100.0, 100.0) | 2.77 (2.41, 3.18) | 0.35 | 2.12 | 2.83 | 3.75 | 8.54 |
|  | Maryland | 32 | 100.0 (100.0, 100.0) | 1.13 (0.94, 1.36) | 0.30 | 0.79 | 1.28 | 1.48 | 3.46 |
|  | Tennessee | 74 | 100.0 (100.0, 100.0) | 1.01 (0.90, 1.13) | 0.23 | 0.76 | 1.07 | 1.41 | 2.87 |
|  | Kansas & Missouri | 52 | 100.0 (100.0, 100.0) | 0.64 (0.57, 0.73) | 0.26 | 0.45 | 0.63 | 0.84 | 2.31 |
|  | Washington | 50 | 98.0 (94.1, 100.0) | 0.76 (0.60, 0.97) | <LOD | 0.53 | 0.85 | 1.16 | 4.03 |
| PFOS | All (550) | 549 | 99.8 (99.5, 100.0) | 2.87 (2.70, 3.05) | <LOD | 1.79 | 2.94 | 4.56 | 52.80 |
|  | Maine - Central | 39 | 100.0 (100.0, 100.0) | 4.15 (3.52, 4.90) | 1.64 | 2.83 | 4.07 | 6.26 | 19.20 |
|  | Maine - South | 45 | 100.0 (100.0, 100.0) | 3.63 (3.16, 4.17) | 0.97 | 2.79 | 3.95 | 4.78 | 9.46 |
|  | Connecticut | 29 | 100.0 (100.0, 100.0) | 3.00 (2.48, 3.64) | 1.35 | 2.02 | 3.00 | 4.33 | 8.11 |
|  | New Jersey - Central | 50 | 100.0 (100.0, 100.0) | 3.33 (2.81, 3.94) | 0.51 | 2.33 | 3.54 | 4.98 | 10.36 |
|  | New Jersey - Coastal | 110 | 100.0 (100.0, 100.0) | 2.64 (2.32, 3.00) | 0.34 | 1.70 | 2.58 | 3.79 | 17.00 |
|  | New Jersey - North | 60 | 100.0 (100.0, 100.0) | 3.58 (3.02, 4.26) | 0.43 | 2.31 | 3.84 | 5.43 | 16.02 |
|  | Maryland | 32 | 100.0 (100.0, 100.0) | 3.57 (2.69, 4.73) | 0.80 | 2.11 | 3.24 | 6.02 | 21.40 |
|  | Tennessee | 74 | 100.0 (100.0, 100.0) | 3.08 (2.65, 3.57) | 0.63 | 2.12 | 3.12 | 4.66 | 15.48 |
|  | Kansas & Missouri | 52 | 100.0 (100.0, 100.0) | 1.79 (1.54, 2.08) | 0.51 | 1.29 | 1.77 | 2.66 | 5.60 |
|  | Washington | 50 | 98.0 (94.1, 100.0) | 1.81 (1.30, 2.52) | <LOD | 1.15 | 1.62 | 2.88 | 52.80 |
| PFNA | All (550) | 549 | 99.8 (99.5, 100.0) | 0.56 (0.54, 0.59) | <LOD | 0.40 | 0.59 | 0.80 | 35.20 |
|  | Maine - Central | 39 | 100.0 (100.0, 100.0) | 0.72 (0.59, 0.87) | 0.32 | 0.50 | 0.69 | 0.87 | 11.30 |
|  | Maine - South | 45 | 100.0 (100.0, 100.0) | 0.75 (0.65, 0.86) | 0.31 | 0.55 | 0.74 | 0.97 | 2.19 |
|  | Connecticut | 29 | 100.0 (100.0, 100.0) | 0.61 (0.53, 0.70) | 0.23 | 0.57 | 0.63 | 0.74 | 1.11 |
|  | New Jersey - Central | 50 | 100.0 (100.0, 100.0) | 0.66 (0.59, 0.75) | 0.19 | 0.49 | 0.69 | 0.88 | 1.51 |
|  | New Jersey - Coastal | 110 | 100.0 (100.0, 100.0) | 0.50 (0.44, 0.56) | 0.03 | 0.35 | 0.52 | 0.77 | 2.37 |
|  | New Jersey - North | 60 | 100.0 (100.0, 100.0) | 0.68 (0.60, 0.78) | 0.14 | 0.58 | 0.68 | 0.85 | 2.86 |
|  | Maryland | 32 | 100.0 (100.0, 100.0) | 0.90 (0.67, 1.21) | 0.26 | 0.60 | 0.81 | 1.19 | 35.20 |
|  | Tennessee | 74 | 100.0 (100.0, 100.0) | 0.58 (0.52, 0.65) | 0.13 | 0.42 | 0.58 | 0.78 | 2.73 |
|  | Kansas & Missouri | 52 | 100.0 (100.0, 100.0) | 0.36 (0.32, 0.40) | 0.16 | 0.27 | 0.35 | 0.49 | 0.88 |
|  | Washington | 50 | 98.0 (94.1, 100.0) | 0.36 (0.28, 0.44) | <LOD | 0.28 | 0.37 | 0.54 | 1.58 |
| PFHxS | All (550) | 549 | 99.8 (99.5, 100.0) | 1.17 (1.09, 1.25) | <LOD | 0.74 | 1.23 | 1.99 | 26.67 |
|  | Maine - Central | 39 | 100.0 (100.0, 100.0) | 0.89 (0.68, 1.17) | 0.15 | 0.46 | 0.80 | 1.59 | 7.23 |
|  | Maine - South | 45 | 100.0 (100.0, 100.0) | 1.29 (1.02, 1.63) | 0.21 | 0.77 | 1.20 | 2.29 | 6.13 |
|  | Connecticut | 29 | 100.0 (100.0, 100.0) | 1.35 (1.09, 1.68) | 0.43 | 0.97 | 1.34 | 1.92 | 4.33 |
|  | New Jersey - Central | 50 | 100.0 (100.0, 100.0) | 1.61 (1.35, 1.93) | 0.17 | 1.27 | 1.65 | 2.27 | 9.44 |
|  | New Jersey - Coastal | 110 | 100.0 (100.0, 100.0) | 1.17 (1.02, 1.33) | 0.19 | 0.82 | 1.16 | 1.81 | 26.67 |
|  | New Jersey - North | 60 | 100.0 (100.0, 100.0) | 2.04 (1.74, 2.39) | 0.09 | 1.56 | 2.24 | 2.83 | 6.12 |
|  | Maryland | 32 | 100.0 (100.0, 100.0) | 1.03 (0.72, 1.46) | 0.14 | 0.59 | 1.04 | 1.98 | 7.92 |
|  | Tennessee | 74 | 100.0 (100.0, 100.0) | 1.09 (0.94, 1.26) | 0.14 | 0.73 | 1.12 | 1.60 | 4.34 |
|  | Kansas & Missouri | 52 | 100.0 (100.0, 100.0) | 0.89 (0.72, 1.10) | 0.16 | 0.50 | 0.88 | 1.49 | 5.23 |
|  | Washington | 50 | 98.0 (94.1, 100.0) | 0.77 (0.58, 1.00) | <LOD | 0.44 | 0.88 | 1.67 | 3.28 |
| PFDA | All (550) | 423 | 76.9 (73.4, 80.4) | 0.07 (0.06, 0.07) | <LOD | 0.02 | 0.08 | 0.16 | 8.76 |
|  | Maine - Central | 21 | 53.8 (37.5, 70.2) | 0.06 (0.04, 0.10) | <LOD | <LOD | 0.11 | 0.16 | 8.76 |
|  | Maine - South | 45 | 100.0 (100.0, 100.0) | 0.13 (0.10, 0.16) | 0.02 | 0.09 | 0.13 | 0.20 | 0.69 |
|  | Connecticut | 29 | 100.0 (100.0, 100.0) | 0.12 (0.10, 0.15) | 0.03 | 0.08 | 0.11 | 0.18 | 0.48 |
|  | New Jersey - Central | 38 | 76.0 (63.7, 88.3) | 0.09 (0.06, 0.12) | <LOD | 0.04 | 0.14 | 0.20 | 0.94 |
|  | New Jersey - Coastal | 41 | 80.4 (69.1, 91.7) | 0.04 (0.03, 0.04) | <LOD | 0.01 | 0.03 | 0.10 | 0.64 |
|  | New Jersey - North | 56 | 93.3 (86.8, 99.8) | 0.10 (0.08, 0.12) | <LOD | 0.06 | 0.12 | 0.19 | 0.49 |
|  | Maryland | 22 | 68.8 (51.8, 85.7) | 0.08 (0.05, 0.13) | <LOD | <LOD | 0.12 | 0.20 | 0.90 |
|  | Tennessee | 64 | 86.5 (78.5, 94.5) | 0.08 (0.06, 0.10) | <LOD | 0.05 | 0.08 | 0.15 | 1.21 |
|  | Kansas & Missouri | 41 | 78.8 (67.4, 90.3) | 0.04 (0.03, 0.05) | <LOD | 0.02 | 0.04 | 0.07 | 0.14 |
|  | Washington | 41 | 80.4 (69.1, 91.7) | 0.06 (0.05, 0.08) | <LOD | 0.03 | 0.06 | 0.12 | 1.16 |
| PFUnDA | All (550) | 422 | 76.7 (73.2, 80.3) | 0.03 (0.03, 0.04) | <LOD | 0.01 | 0.05 | 0.10 | 0.82 |
|  | Maine - Central | 23 | 59.0 (42.8, 75.1) | 0.02 (0.01, 0.04) | <LOD | <LOD | 0.04 | 0.10 | 0.66 |
|  | Maine - South | 43 | 95.6 (89.3, 100.0) | 0.07 (0.05, 0.10) | <LOD | 0.04 | 0.10 | 0.13 | 0.82 |
|  | Connecticut | 28 | 96.6 (89.5, 100.0) | 0.08 (0.05, 0.11) | <LOD | 0.05 | 0.08 | 0.15 | 0.36 |
|  | New Jersey - Central | 42 | 84.0 (73.5, 94.5) | 0.06 (0.04, 0.08) | <LOD | 0.03 | 0.09 | 0.13 | 0.46 |
|  | New Jersey - Coastal | 60 | 54.5 (45.1, 64.0) | 0.02 (0.01, 0.03) | <LOD | <LOD | 0.02 | 0.07 | 0.61 |
|  | New Jersey - North | 52 | 86.7 (77.8, 95.5) | 0.05 (0.04, 0.08) | <LOD | 0.04 | 0.08 | 0.13 | 0.35 |
|  | Maryland | 31 | 96.9 (90.5, 100.0) | 0.08 (0.06, 0.12) | <LOD | 0.06 | 0.07 | 0.16 | 0.44 |
|  | Tennessee | 70 | 94.6 (89.3, 99.9) | 0.04 (0.03, 0.05) | <LOD | 0.02 | 0.04 | 0.08 | 0.60 |
|  | Kansas & Missouri | 33 | 63.5 (49.9, 77.0) | 0.01 (0.01, 0.02) | <LOD | <LOD | 0.01 | 0.03 | 0.10 |
|  | Washington | 34 | 66.7 (53.3, 80.1) | 0.02 (0.02, 0.03) | <LOD | <LOD | 0.03 | 0.07 | 0.33 |
| MeFOSAA | All (550) | 260 | 47.3 (43.1, 51.5) | <LOD | <LOD | <LOD | <LOD | 0.04 | 3.77 |
|  | Maine - Central | 12 | 30.8 (15.6, 45.9) |  | <LOD | <LOD | <LOD | 0.04 | 0.32 |
|  | Maine - South | 30 | 66.7 (52.3, 81.0) |  | <LOD | <LOD | 0.02 | 0.04 | 0.20 |
|  | Connecticut | 23 | 79.3 (63.6, 95.0) |  | <LOD | 0.01 | 0.03 | 0.07 | 0.22 |
|  | New Jersey - Central | 19 | 38.0 (24.1, 51.9) |  | <LOD | <LOD | <LOD | 0.03 | 1.15 |
|  | New Jersey - Coastal | 40 | 36.4 (27.2, 45.5) |  | <LOD | <LOD | <LOD | 0.05 | 1.04 |
|  | New Jersey - North | 24 | 40.0 (27.2, 52.8) |  | <LOD | <LOD | <LOD | 0.04 | 0.64 |
|  | Maryland | 17 | 53.1 (34.8, 71.4) |  | <LOD | <LOD | 0.02 | 0.07 | 1.08 |
|  | Tennessee | 40 | 54.1 (42.4, 65.7) |  | <LOD | <LOD | 0.01 | 0.05 | 0.76 |
|  | Kansas & Missouri | 27 | 51.9 (37.9, 66.0) |  | <LOD | <LOD | 0.01 | 0.04 | 0.30 |
|  | Washington | 22 | 43.1 (29.1, 57.2) |  | <LOD | <LOD | <LOD | 0.03 | 3.77 |
| PFHpA | All (550) | 193 | 35.1 (31.1, 39.1) | <LOD | <LOD | <LOD | <LOD | 0.04 | 0.71 |
|  | Maine - Central | 15 | 38.5 (22.5, 54.4) |  | <LOD | <LOD | <LOD | 0.08 | 0.21 |
|  | Maine - South | 30 | 66.7 (52.3, 81.0) |  | <LOD | <LOD | 0.03 | 0.07 | 0.19 |
|  | Connecticut | 15 | 51.7 (32.4, 71.1) |  | <LOD | <LOD | 0.01 | 0.05 | 0.16 |
|  | New Jersey - Central | 18 | 36.0 (22.2, 49.8) |  | <LOD | <LOD | <LOD | 0.07 | 0.71 |
|  | New Jersey - Coastal | 25 | 22.7 (14.8, 30.7) |  | <LOD | <LOD | <LOD | <LOD | 0.21 |
|  | New Jersey - North | 25 | 41.7 (28.8, 54.5) |  | <LOD | <LOD | <LOD | 0.06 | 0.22 |
|  | Maryland | 12 | 37.5 (19.8, 55.2) |  | <LOD | <LOD | <LOD | 0.04 | 0.14 |
|  | Tennessee | 30 | 40.5 (29.1, 52.0) |  | <LOD | <LOD | <LOD | 0.04 | 0.17 |
|  | Kansas & Missouri | 14 | 26.9 (14.5, 39.4) |  | <LOD | <LOD | <LOD | 0.01 | 0.14 |
|  | Washington | 6 | 11.8 (2.6, 20.9) |  | <LOD | <LOD | <LOD | <LOD | 0.33 |
| FOSA | All (550) | 127 | 23.1 (19.6, 26.6) | <LOD | <LOD | <LOD | <LOD | <LOD | 0.19 |
|  | Maine - Central | 2 | 5.1 (0.0, 12.4) |  | <LOD | <LOD | <LOD | <LOD | 0.08 |
|  | Maine - South | 13 | 28.9 (15.1, 42.7) |  | <LOD | <LOD | <LOD | 0.00 | 0.03 |
|  | Connecticut | 17 | 58.6 (39.6, 77.7) |  | <LOD | <LOD | 0.01 | 0.01 | 0.04 |
|  | New Jersey - Central | 11 | 22.0 (10.1, 33.9) |  | <LOD | <LOD | <LOD | <LOD | 0.10 |
|  | New Jersey - Coastal | 29 | 26.4 (18.0, 34.7) |  | <LOD | <LOD | <LOD | 0.00 | 0.10 |
|  | New Jersey - North | 19 | 31.7 (19.5, 43.8) |  | <LOD | <LOD | <LOD | 0.01 | 0.15 |
|  | Maryland | 4 | 12.5 (0.4, 24.6) |  | <LOD | <LOD | <LOD | <LOD | 0.11 |
|  | Tennessee | 19 | 25.7 (15.5, 35.9) |  | <LOD | <LOD | <LOD | 0.00 | 0.19 |
|  | Kansas & Missouri | 5 | 9.6 (1.3, 17.9) |  | <LOD | <LOD | <LOD | <LOD | 0.04 |
|  | Washington | 6 | 11.8 (2.6, 20.9) |  | <LOD | <LOD | <LOD | <LOD | 0.12 |
| PFBS | All (550) | 67 | 12.2 (9.4, 14.9) | <LOD | <LOD | <LOD | <LOD | <LOD | 1.78 |
|  | Maine - Central | 0 | 0.0 (0.0, 0.0) |  | <LOD | <LOD | <LOD | <LOD | <LOD |
|  | Maine - South | 0 | 0.0 (0.0, 0.0) |  | <LOD | <LOD | <LOD | <LOD | <LOD |
|  | Connecticut | 1 | 3.4 (0.0, 10.5) |  | <LOD | <LOD | <LOD | <LOD | 0.99 |
|  | New Jersey - Central | 12 | 24.0 (11.7, 36.3) |  | <LOD | <LOD | <LOD | <LOD | 0.13 |
|  | New Jersey - Coastal | 0 | 0.0 (0.0, 0.0) |  | <LOD | <LOD | <LOD | <LOD | <LOD |
|  | New Jersey - North | 11 | 18.3 (8.3, 28.4) |  | <LOD | <LOD | <LOD | <LOD | 0.10 |
|  | Maryland | 1 | 3.1 (0.0, 9.5) |  | <LOD | <LOD | <LOD | <LOD | 0.08 |
|  | Tennessee | 1 | 1.4 (0.0, 4.0) |  | <LOD | <LOD | <LOD | <LOD | 1.78 |
|  | Kansas & Missouri | 0 | 0.0 (0.0, 0.0) |  | <LOD | <LOD | <LOD | <LOD | <LOD |
|  | Washington | 41 | 80.4 (69.1, 91.7) |  | <LOD | 0.01 | 0.03 | 0.05 | 0.33 |
| PFDoDA | All (550) | 62 | 11.3 (8.6, 13.9) | <LOD | <LOD | <LOD | <LOD | <LOD | 0.21 |
|  | Maine - Central | 1 | 2.6 (0.0, 7.8) |  | <LOD | <LOD | <LOD | <LOD | 0.13 |
|  | Maine - South | 13 | 28.9 (15.1, 42.7) |  | <LOD | <LOD | <LOD | 0.02 | 0.14 |
|  | Connecticut | 10 | 34.5 (16.1, 52.9) |  | <LOD | <LOD | <LOD | 0.02 | 0.05 |
|  | New Jersey - Central | 7 | 14.0 (4.0, 24.0) |  | <LOD | <LOD | <LOD | <LOD | 0.15 |
|  | New Jersey - Coastal | 6 | 5.5 (1.1, 9.8) |  | <LOD | <LOD | <LOD | <LOD | 0.07 |
|  | New Jersey - North | 9 | 15.0 (5.7, 24.3) |  | <LOD | <LOD | <LOD | <LOD | 0.11 |
|  | Maryland | 3 | 9.4 (0.0, 20.1) |  | <LOD | <LOD | <LOD | <LOD | 0.16 |
|  | Tennessee | 7 | 9.5 (2.6, 16.3) |  | <LOD | <LOD | <LOD | <LOD | 0.16 |
|  | Kansas & Missouri | 0 | 0.0 (0.0, 0.0) |  | <LOD | <LOD | <LOD | <LOD | <LOD |
|  | Washington | 6 | 11.8 (2.6, 20.9) |  | <LOD | <LOD | <LOD | <LOD | 0.21 |
| EtFOSAA | All (550) | 29 | 5.3 (3.4, 7.1) | <LOD | <LOD | <LOD | <LOD | <LOD | 0.19 |
|  | Maine - Central | 1 | 2.6 (0.0, 7.8) |  | <LOD | <LOD | <LOD | <LOD | 0.19 |
|  | Maine - South | 5 | 11.1 (1.6, 20.7) |  | <LOD | <LOD | <LOD | <LOD | 0.14 |
|  | Connecticut | 5 | 17.2 (2.6, 31.9) |  | <LOD | <LOD | <LOD | <LOD | 0.10 |
|  | New Jersey - Central | 1 | 2.0 (0.0, 6.0) |  | <LOD | <LOD | <LOD | <LOD | 0.05 |
|  | New Jersey - Coastal | 4 | 3.6 (0.1, 7.2) |  | <LOD | <LOD | <LOD | <LOD | 0.02 |
|  | New Jersey - North | 4 | 6.7 (0.2, 13.2) |  | <LOD | <LOD | <LOD | <LOD | 0.18 |
|  | Maryland | 1 | 3.1 (0.0, 9.5) |  | <LOD | <LOD | <LOD | <LOD | 0.01 |
|  | Tennessee | 5 | 6.8 (0.9, 12.6) |  | <LOD | <LOD | <LOD | <LOD | 0.05 |
|  | Kansas & Missouri | 3 | 5.8 (0.0, 12.3) |  | <LOD | <LOD | <LOD | <LOD | 0.05 |
|  | Washington | 0 | 0.0 (0.0, 0.0) |  | <LOD | <LOD | <LOD | <LOD | <LOD |
| **Notes**: CAPS: Cancer Assessment and Prevention Study; DF: detection frequency; CI: confidence interval; GM: geometric mean; Min: minimum; Q1, Q2, Q3: first, second, and third quartile; Max: maximum; LOD: limit of detection; PFOA: perfluorooctanoic acid; PFOS: perfluorooctane sulfonic acid; PFNA: perfluorononanoic acid; PFHxS: perfluorohexane sulfonic acid; PFDA: perfluorodecanoic acid ; PFUnDA: perfluoroundecanoic acid; MeFOSAA: 2-(n-methyl-perfluorooctane sulfonamido) acetic acid; PFHpA: perfluoroheptanoic acid; FOSA: perfluorooctane sulfonamide; PFBS: perfluorobutane sulfonic acid; PFDoDA: perfluorododecanoic acid; EtFOSAA: 2-(n-ethyl-perfluorooctane sulfonamido) acetic acid. | | | | | | | | | |

| **Supplemental Table 5. Pearson correlations between ln-transformed serum PFAS detected in >75% of CAPS participants (n = 550).** | | | | | | |
| --- | --- | --- | --- | --- | --- | --- |
|  | ln-PFOA | ln-PFOS | ln-PFNA | ln-PFHxS | ln-PFDA | ln-PFUnDA |
| ln-PFOA | 1.00 | 0.642 | 0.609 | 0.668 | 0.368 | 0.349 |
|  |  | <.0001 | <.0001 | <.0001 | <.0001 | <.0001 |
| ln-PFOS |  | 1.00 | 0.728 | 0.679 | 0.570 | 0.513 |
|  |  |  | <.0001 | <.0001 | <.0001 | <.0001 |
| ln-PFNA |  |  | 1.00 | 0.523 | 0.544 | 0.518 |
|  |  |  |  | <.0001 | <.0001 | <.0001 |
| ln-PFHxS |  |  |  | 1.00 | 0.391 | 0.295 |
|  |  |  |  |  | <.0001 | <.0001 |
| ln-PFDA |  |  |  |  | 1.00 | 0.771 |
|  |  |  |  |  |  | <.0001 |
| ln-PFUnDA |  |  |  |  |  | 1.00 |
|  |  |  |  |  |  |  |
| Notes: The first number for each pair indicates the Pearson correlation coefficient, and the second number for each pair shows the p-value (prob > \|r\| under H0: rho = 0) | | | | | | |

| **Supplemental Table 6. Multivariable associations between PFAS and predictors of interest, using GEE (proc genmod) linear (ln-PFAS) and logistic (detected vs. not detected) regression models (n = 550)** | | | | | | | | | | |
| --- | --- | --- | --- | --- | --- | --- | --- | --- | --- | --- |
|  | **ln-PFOA** | **ln-PFOS** | **ln-PFNA** | **ln-PFHxS** | **ln-PFDA** | **ln-PFUnDA** | **MeFOSAA** | **PFHpA** | **FOSA** |  |
| **Characteristic** | β (95% CI) | β (95% CI) | β (95% CI) | β (95% CI) | β (95% CI) | β (95% CI) | OR (95% CI) | OR (95% CI) | OR (95% CI) |  |
| **Sociodemographic** |  |  |  |  |  |  |  |  |  |  |
| Age (10 yrs) | 0.04 (-0.01, 0.08) | 0.09 (0.02, 0.15) | 0.06 (0.01, 0.11) | 0.04 (-0.02, 0.11) | 0.12 (0.03, 0.21) | 0.20 (0.08, 0.32) | 1.07 (0.90, 1.26) | 1.07 (0.87, 1.31) | 1.29 (1.00, 1.67) |  |
| Sex (ref: female) | 0.32 (0.14, 0.50) | 0.35 (0.17, 0.54) | 0.35 (0.16, 0.54) | 0.65 (0.44, 0.86) | 0.10 (-0.14, 0.34) | 0.02 (-0.29, 0.34) | 0.86 (0.46, 1.62) | 1.00 (0.57, 1.75) | 0.46 (0.27, 0.79) |  |
| Education (ref: ≤High school) |  |  |  |  |  |  |  |  |  |  |
| Some college, AA, technical | 0.03 (-0.07, 0.13) | -0.01 (-0.13, 0.10) | 0.03 (-0.05, 0.11) | -0.01 (-0.14, 0.13) | 0.17 (-0.03, 0.37) | 0.23 (0.00, 0.46) | 0.88 (0.54, 1.45) | 0.71 (0.45, 1.11) | 1.00 (0.59, 1.69) |  |
| ≥Bachelor's degree | 0.23 (0.10, 0.35) | 0.17 (0.02, 0.32) | 0.10 (-0.06, 0.26) | 0.23 (0.05, 0.42) | 0.53 (0.29, 0.77) | 0.60 (0.33, 0.87) | 1.42 (0.83, 2.45) | 0.88 (0.55, 1.42) | 0.90 (0.51, 1.59) |  |
| Income (ref: <$50,000) |  |  |  |  |  |  |  |  |  |  |
| $50,000 - <$75,000 | 0.09 (-0.09, 0.26) | 0.11 (-0.08, 0.30) | 0.09 (-0.07, 0.25) | 0.11 (-0.10, 0.33) | 0.31 (0.05, 0.58) | 0.28 (-0.07, 0.62) | 0.97 (0.56, 1.70) | 1.38 (0.77, 2.50) | 0.94 (0.43, 2.08) |  |
| $75,000 - <$100,000 | 0.19 (0.04, 0.35) | 0.27 (0.06, 0.47) | 0.26 (0.11, 0.42) | 0.29 (0.07, 0.50) | 0.37 (0.08, 0.66) | 0.40 (0.04, 0.76) | 0.64 (0.37, 1.09) | 1.75 (0.92, 3.30) | 1.44 (0.73, 2.86) |  |
| $100,000 - <$150,000 | 0.14 (-0.01, 0.29) | 0.10 (-0.08, 0.28) | 0.09 (-0.05, 0.23) | 0.18 (-0.04, 0.41) | 0.15 (-0.10, 0.40) | 0.11 (-0.24, 0.47) | 0.67 (0.37, 1.23) | 1.65 (0.96, 2.82) | 1.00 (0.48, 2.12) |  |
| >$150,000 | 0.08 (-0.07, 0.23) | 0.12 (-0.04, 0.28) | 0.14 (-0.03, 0.30) | 0.13 (-0.08, 0.33) | 0.28 (0.03, 0.53) | 0.35 (-0.01, 0.71) | 0.73 (0.40, 1.33) | 1.16 (0.63, 2.12) | 0.98 (0.44, 2.19) |  |
| Hazardous drinking (ref: no) | 0.12 (0.03, 0.21) | 0.00 (-0.11, 0.12) | 0.02 (-0.06, 0.10) | 0.00 (-0.11, 0.11) | 0.13 (-0.04, 0.31) | 0.51 (0.25, 0.76) | 1.12 (0.76, 1.65) | 1.50 (0.99, 2.25) | 1.08 (0.70, 1.67) |  |
| Cigarette use (ref: never) |  |  |  |  |  |  |  |  |  |  |
| Former | 0.10 (-0.04, 0.23) | 0.22 (0.04, 0.41) | 0.08 (-0.04, 0.21) | 0.19 (0.03, 0.35) | 0.18 (-0.10, 0.47) | 0.04 (-0.27, 0.35) | 0.96 (0.58, 1.58) | 1.38 (0.84, 2.27) | 0.84 (0.45, 1.56) |  |
| Current | -0.03 (-0.18, 0.11) | 0.10 (-0.05, 0.26) | 0.09 (-0.09, 0.27) | 0.09 (-0.08, 0.27) | -0.01 (-0.33, 0.30) | -0.06 (-0.47, 0.35) | 0.98 (0.52, 1.84) | 0.92 (0.43, 1.93) | 0.94 (0.48, 1.85) |  |
| Pesticide/herbicide use (ref: no) | 0.03 (-0.06, 0.12) | 0.09 (-0.01, 0.18) | -0.01 (-0.09, 0.08) | 0.05 (-0.07, 0.16) | 0.09 (-0.05, 0.23) | 0.07 (-0.12, 0.26) | 1.09 (0.79, 1.52) | 0.69 (0.48, 1.00) | 0.95 (0.62, 1.46) |  |
| **Occupational** |  |  |  |  |  |  |  |  |  |  |
| Military service (ref: no) | 0.05 (-0.10, 0.21) | 0.04 (-0.14, 0.22) | 0.05 (-0.10, 0.21) | 0.09 (-0.11, 0.30) | 0.13 (-0.10, 0.37) | 0.17 (-0.14, 0.47) | 0.77 (0.40, 1.49) | 1.23 (0.69, 2.20) | 1.40 (0.78, 2.53) |  |
| Primary occupation (ref: not employed) |  |  |  |  |  |  |  |  |  |  |
| Construction | -0.08 (-0.26, 0.10) | 0.01 (-0.14, 0.17) | 0.01 (-0.13, 0.14) | 0.00 (-0.20, 0.21) | 0.05 (-0.21, 0.31) | 0.07 (-0.30, 0.43) | 0.85 (0.44, 1.66) | 1.38 (0.71, 2.68) | 0.79 (0.37, 1.71) |  |
| Fire Protection | -0.09 (-0.30, 0.13) | -0.04 (-0.25, 0.17) | -0.14 (-0.32, 0.05) | 0.02 (-0.23, 0.27) | -0.31 (-0.69, 0.06) | -0.36 (-0.87, 0.14) | 1.58 (0.58, 4.35) | 0.57 (0.26, 1.26) | 0.78 (0.25, 2.40) |  |
| Professional | -0.08 (-0.26, 0.11) | 0.00 (-0.22, 0.22) | 0.01 (-0.16, 0.17) | 0.11 (-0.13, 0.36) | 0.08 (-0.31, 0.47) | -0.05 (-0.51, 0.40) | 0.52 (0.24, 1.13) | 1.28 (0.61, 2.68) | 0.79 (0.35, 1.75) |  |
| Public Admin, Public Order | -0.15 (-0.34, 0.04) | -0.16 (-0.38, 0.07) | 0.01 (-0.18, 0.19) | -0.05 (-0.26, 0.16) | -0.16 (-0.46, 0.15) | -0.11 (-0.51, 0.29) | 0.98 (0.48, 2.00) | 1.22 (0.63, 2.36) | 0.93 (0.43, 2.02) |  |
| Retail, Health Care | -0.11 (-0.35, 0.13) | -0.15 (-0.37, 0.08) | -0.12 (-0.34, 0.09) | -0.21 (-0.42, 0.00) | -0.09 (-0.48, 0.30) | -0.17 (-0.59, 0.24) | 0.97 (0.43, 2.16) | 1.17 (0.50, 2.75) | 0.57 (0.23, 1.40) |  |
| Other Services | -0.24 (-0.42, -0.06) | -0.21 (-0.41, 0.00) | -0.24 (-0.40, -0.07) | -0.23 (-0.43, -0.03) | -0.28 (-0.63, 0.07) | -0.43 (-0.92, 0.06) | 0.64 (0.33, 1.23) | 1.28 (0.65, 2.54) | 0.69 (0.33, 1.43) |  |
| Transportation | -0.20 (-0.39, 0.00) | -0.15 (-0.37, 0.07) | -0.14 (-0.31, 0.03) | -0.29 (-0.56, -0.02) | -0.24 (-0.52, 0.03) | -0.48 (-0.89, -0.07) | 1.07 (0.53, 2.19) | 2.26 (1.03, 4.95) | 0.84 (0.36, 1.95) |  |
| **Fire service** |  |  |  |  |  |  |  |  |  |  |
| Years of vol. ff experience (10 yrs) | 0.01 (-0.04, 0.06) | 0.04 (-0.02, 0.10) | -0.02 (-0.08, 0.03) | 0.01 (-0.06, 0.08) | -0.03 (-0.12, 0.07) | -0.05 (-0.18, 0.08) | 1.06 (0.89, 1.27) | 1.08 (0.88, 1.34) | 1.13 (0.93, 1.36) |  |
| Career firefighter (ref: never) |  |  |  |  |  |  |  |  |  |  |
| Former | -0.02 (-0.21, 0.18) | 0.14 (-0.12, 0.41) | 0.08 (-0.16, 0.31) | 0.23 (-0.07, 0.52) | 0.09 (-0.39, 0.58) | 0.02 (-0.60, 0.65) | 1.80 (0.65, 4.98) | 0.89 (0.44, 1.79) | 0.87 (0.42, 1.82) |  |
| Current | -0.04 (-0.23, 0.14) | 0.14 (-0.05, 0.32) | 0.07 (-0.09, 0.23) | 0.06 (-0.16, 0.28) | 0.28 (-0.12, 0.69) | 0.24 (-0.17, 0.66) | 0.85 (0.39, 1.82) | 1.28 (0.56, 2.91) | 0.68 (0.27, 1.72) |  |
| Monthly calls (ref:< 5) |  |  |  |  |  |  |  |  |  |  |
| 5 - 10 calls | -0.03 (-0.19, 0.13) | 0.07 (-0.11, 0.24) | 0.15 (0.02, 0.28) | 0.05 (-0.14, 0.23) | 0.04 (-0.22, 0.31) | 0.03 (-0.30, 0.37) | 1.26 (0.78, 2.02) | 1.12 (0.69, 1.83) | 0.99 (0.51, 1.93) |  |
| 11 - 20 calls | 0.04 (-0.12, 0.20) | 0.10 (-0.07, 0.26) | 0.12 (-0.01, 0.26) | 0.08 (-0.13, 0.28) | 0.04 (-0.23, 0.30) | -0.18 (-0.53, 0.17) | 1.57 (0.88, 2.81) | 0.95 (0.50, 1.80) | 1.16 (0.59, 2.27) |  |
| > 20 calls | 0.06 (-0.10, 0.23) | 0.05 (-0.16, 0.26) | 0.15 (-0.02, 0.32) | 0.16 (-0.04, 0.36) | 0.19 (-0.15, 0.52) | -0.04 (-0.45, 0.38) | 1.28 (0.64, 2.57) | 1.46 (0.73, 2.93) | 1.19 (0.49, 2.88) |  |
| Current rank (ref: FF) |  |  |  |  |  |  |  |  |  |  |
| Firefighter-EMT | 0.00 (-0.16, 0.16) | 0.05 (-0.14, 0.25) | 0.17 (0.00, 0.34) | 0.00 (-0.21, 0.20) | 0.13 (-0.14, 0.39) | 0.08 (-0.23, 0.39) | 1.63 (0.94, 2.83) | 1.23 (0.73, 2.09) | 1.10 (0.55, 2.22) |  |
| Driver Operator / Engineer | 0.09 (-0.06, 0.24) | 0.23 (0.07, 0.39) | 0.15 (-0.02, 0.32) | 0.06 (-0.14, 0.27) | 0.36 (-0.09, 0.81) | 0.26 (-0.30, 0.81) | 3.88 (2.01, 7.50) | 0.93 (0.32, 2.65) | 2.18 (0.79, 6.07) |  |
| Officers | -0.03 (-0.17, 0.12) | -0.01 (-0.16, 0.13) | 0.01 (-0.12, 0.14) | -0.02 (-0.22, 0.18) | 0.03 (-0.29, 0.34) | -0.02 (-0.35, 0.30) | 1.14 (0.70, 1.87) | 0.62 (0.37, 1.05) | 0.58 (0.31, 1.08) |  |
| All Chiefs | 0.06 (-0.13, 0.24) | 0.03 (-0.17, 0.22) | 0.12 (-0.07, 0.30) | 0.05 (-0.21, 0.31) | 0.07 (-0.24, 0.38) | 0.11 (-0.27, 0.48) | 0.83 (0.44, 1.55) | 0.84 (0.47, 1.50) | 0.93 (0.45, 1.94) |  |
| Aux + Recruit | -0.04 (-0.30, 0.23) | -0.15 (-0.48, 0.18) | -0.04 (-0.25, 0.18) | -0.04 (-0.33, 0.24) | -0.08 (-0.35, 0.20) | -0.20 (-0.59, 0.18) | 0.90 (0.47, 1.71) | 0.61 (0.31, 1.19) | 0.79 (0.33, 1.87) |  |
| Years in current rank (ref: < 2 years) |  |  |  |  |  |  |  |  |  |  |
| 2 - 4 years | 0.01 (-0.11, 0.13) | 0.03 (-0.11, 0.18) | 0.05 (-0.06, 0.15) | 0.02 (-0.14, 0.17) | -0.02 (-0.25, 0.21) | -0.20 (-0.49, 0.10) | 0.68 (0.39, 1.19) | 1.17 (0.76, 1.79) | 0.83 (0.44, 1.59) |  |
| 5 - 10 years | 0.02 (-0.13, 0.16) | 0.11 (-0.07, 0.30) | 0.10 (-0.03, 0.22) | 0.17 (0.03, 0.32) | 0.04 (-0.23, 0.30) | -0.04 (-0.41, 0.34) | 0.93 (0.58, 1.47) | 1.10 (0.72, 1.69) | 1.33 (0.76, 2.31) |  |
| > 10 years | -0.06 (-0.19, 0.06) | 0.00 (-0.13, 0.13) | -0.03 (-0.15, 0.09) | 0.00 (-0.14, 0.14) | -0.06 (-0.31, 0.19) | -0.14 (-0.45, 0.16) | 0.70 (0.39, 1.25) | 0.76 (0.46, 1.24) | 0.70 (0.38, 1.31) |  |
| FF tasks categories (ref: Support) |  |  |  |  |  |  |  |  |  |  |
| Technical | 0.16 (0.00, 0.32) | 0.15 (-0.03, 0.34) | -0.01 (-0.15, 0.13) | 0.21 (0.00, 0.43) | 0.17 (-0.11, 0.45) | -0.02 (-0.43, 0.39) | 1.46 (0.73, 2.92) | 0.53 (0.25, 1.14) | 2.02 (1.00, 4.09) |  |
| General | 0.02 (-0.12, 0.16) | 0.04 (-0.09, 0.18) | -0.06 (-0.18, 0.07) | -0.01 (-0.20, 0.19) | -0.07 (-0.34, 0.20) | -0.11 (-0.46, 0.23) | 0.85 (0.47, 1.55) | 0.93 (0.55, 1.59) | 1.24 (0.69, 2.23) |  |
| Interior & medical | 0.01 (-0.14, 0.15) | 0.05 (-0.13, 0.24) | -0.02 (-0.17, 0.14) | 0.02 (-0.18, 0.23) | 0.08 (-0.30, 0.46) | -0.05 (-0.46, 0.37) | 0.96 (0.48, 1.92) | 0.64 (0.36, 1.14) | 1.36 (0.63, 2.92) |  |
| Some on-scene | 0.06 (-0.10, 0.23) | 0.18 (0.02, 0.35) | 0.04 (-0.11, 0.18) | 0.05 (-0.16, 0.27) | 0.16 (-0.10, 0.42) | 0.17 (-0.16, 0.50) | 1.11 (0.65, 1.89) | 0.99 (0.54, 1.81) | 1.30 (0.65, 2.61) |  |
| **Notes**: Current rank reference category is firefighter and includes investigators and instructors. Occupation: Construction also includes agriculture, farming, fishing, mining, and building services; Professional includes finance, education, and information; Retail, Health Care also includes arts and hospitality; Transportation also includes wholesale trade and manufacturing. FF task classes from latent class analysis (see Supplemental Table 3, Supplemental Figure 2) | | | | | | | | | | |

| **Supplemental Table 7.** Multivariable associations between PFAS and predictors of interest, using GEE (proc genmod) linear (ln-PFAS) regression models among male volunteer firefighters under age 50 (n = 306) | | | | | | |
| --- | --- | --- | --- | --- | --- | --- |
|  | **ln-PFOA** | **ln-PFOS** | **ln-PFNA** | **ln-PFHxS** | **ln-PFDA** | **ln-PFUnDA** |
| **Characteristic** | β (95% CI) | β (95% CI) | β (95% CI) | β (95% CI) | β (95% CI) | β (95% CI) |
| **Sociodemographic** |  |  |  |  |  |  |
| Age (10 yrs) | 0.13 (0.03, 0.23) | 0.19 (0.09, 0.29) | 0.10 (0.01, 0.18) | 0.20 (0.07, 0.34) | 0.14 (-0.03, 0.32) | 0.06 (-0.13, 0.25) |
| Education (ref: ≤High school) |  |  |  |  |  |  |
| Some college, AA, tech. | 0.12 (0.01, 0.24) | 0.10 (-0.03, 0.24) | 0.06 (-0.04, 0.16) | 0.07 (-0.09, 0.24) | 0.28 (0.06, 0.51) | 0.23 (-0.03, 0.49) |
| ≥Bachelor's degree | 0.23 (0.12, 0.35) | 0.30 (0.14, 0.46) | 0.16 (0.03, 0.28) | 0.33 (0.15, 0.51) | 0.67 (0.36, 0.98) | 0.61 (0.29, 0.94) |
| HH income (ref: <$50,000) |  |  |  |  |  |  |
| $50,000 - <$100,000 | 0.10 (-0.05, 0.25) | 0.19 (0.01, 0.37) | 0.17 (0.04, 0.30) | 0.14 (-0.06, 0.35) | 0.48 (0.22, 0.74) | 0.58 (0.20, 0.95) |
| ≥$100,000 | 0.02 (-0.17, 0.21) | 0.05 (-0.13, 0.23) | 0.08 (-0.06, 0.23) | 0.06 (-0.18, 0.30) | 0.20 (-0.06, 0.46) | 0.40 (0.03, 0.77) |
| Hazardous drinking (ref: no) | 0.16 (0.05, 0.28) | -0.04 (-0.18, 0.11) | 0.02 (-0.08, 0.12) | -0.02 (-0.18, 0.15) | 0.24 (0.02, 0.46) | 0.68 (0.36, 1.00) |
| Cigarette use (ref: never) |  |  |  |  |  |  |
| Former | 0.01 (-0.14, 0.16) | 0.20 (0.01, 0.39) | 0.14 (0.03, 0.25) | 0.16 (-0.04, 0.36) | 0.05 (-0.29, 0.39) | -0.12 (-0.61, 0.37) |
| Current | -0.08 (-0.27, 0.12) | 0.12 (-0.06, 0.30) | 0.07 (-0.12, 0.26) | 0.06 (-0.15, 0.27) | 0.05 (-0.37, 0.48) | 0.12 (-0.48, 0.71) |
| Pesticide/herbicide use (ref: no) | 0.00 (-0.09, 0.10) | 0.09 (-0.01, 0.19) | 0.03 (-0.06, 0.13) | 0.06 (-0.08, 0.20) | 0.05 (-0.15, 0.25) | -0.10 (-0.35, 0.15) |
| **Occupational** |  |  |  |  |  |  |
| Employed ≥ 35 hrs/week (ref: work < 35 hrs/wk) | -0.06 (-0.27, 0.14) | -0.04 (-0.31, 0.22) | -0.04 (-0.24, 0.17) | -0.04 (-0.35, 0.27) | -0.24 (-0.65, 0.18) | -0.14 (-0.70, 0.43) |
| **Fire service** |  |  |  |  |  |  |
| Years of vol. FF experience (10 yrs) | 0.05 (-0.04, 0.14) | 0.05 (-0.06, 0.15) | 0.03 (-0.06, 0.11) | -0.01 (-0.14, 0.11) | 0.04 (-0.14, 0.22) | 0.08 (-0.13, 0.30) |
| Ever career FF (ref: no) | -0.02 (-0.13, 0.10) | 0.06 (-0.08, 0.21) | 0.01 (-0.15, 0.17) | 0.22 (0.03, 0.42) | 0.02 (-0.31, 0.35) | 0.06 (-0.38, 0.49) |
| Monthly calls (ref:< 5) |  |  |  |  |  |  |
| 5 - 14 calls | -0.10 (-0.27, 0.07) | -0.01 (-0.19, 0.18) | 0.11 (-0.04, 0.26) | -0.03 (-0.27, 0.22) | 0.07 (-0.25, 0.39) | 0.04 (-0.32, 0.40) |
| ≥ 15 calls | -0.04 (-0.19, 0.10) | 0.00 (-0.17, 0.17) | 0.11 (-0.03, 0.25) | 0.06 (-0.15, 0.27) | 0.18 (-0.14, 0.50) | -0.19 (-0.67, 0.29) |
| Notes. Beta estimates and 95% confidence intervals (CIs) represent the change in ln-transformed PFAS per change in the independent variable, adjusted for the other factors shown and accounting for clustering within fire departments. CAPS: Cancer Assessment and Prevention Study; FF: firefighter. | | | | | | |

| **Supplemental Table 8.** Multivariable associations between PFAS and predictors of interest, using GEE (proc genmod) linear (ln-PFAS) regression models among female volunteer firefighters under age 50 (n = 54) | | | | | | |
| --- | --- | --- | --- | --- | --- | --- |
|  | **ln-PFOA** | **ln-PFOS** | **ln-PFNA** | **ln-PFHxS** | **ln-PFDA** | **ln-PFUnDA** |
| **Characteristic** | β (95% CI) | β (95% CI) | β (95% CI) | β (95% CI) | β (95% CI) | β (95% CI) |
| **Sociodemographic** |  |  |  |  |  |  |
| Age (10 yrs) | -0.05 (-0.32, 0.22) | 0.21 (-0.27, 0.69) | -0.05 (-0.37, 0.27) | 0.13 (-0.31, 0.57) | -0.39 (-1.47, 0.70) | -0.32 (-1.57, 0.92) |
| Education (ref: ≤High school) |  |  |  |  |  |  |
| Some college, AA, tech. | 0.25 (-0.48, 0.98) | -0.06 (-1.06, 0.93) | 0.10 (-0.53, 0.73) | 0.00 (-0.76, 0.75) | 0.47 (-0.81, 1.75) | 0.57 (-0.98, 2.12) |
| ≥Bachelor's degree | 0.68 (0.04, 1.32) | 0.34 (-0.64, 1.32) | 0.41 (-0.21, 1.02) | 0.32 (-0.28, 0.93) | 1.39 (0.66, 2.13) | 2.32 (0.86, 3.77) |
| HH income (ref: <$50,000) |  |  |  |  |  |  |
| $50,000 - <$100,000 | -0.13 (-0.81, 0.54) | -0.08 (-0.99, 0.83) | 0.04 (-0.57, 0.66) | -0.15 (-0.92, 0.63) | -0.37 (-1.35, 0.61) | -0.85 (-2.56, 0.87) |
| ≥$100,000 | 0.38 (-0.14, 0.90) | 0.35 (-0.32, 1.03) | 0.42 (0.00, 0.83) | 0.44 (-0.14, 1.03) | 0.64 (-0.09, 1.37) | 0.30 (-0.76, 1.36) |
| Hazardous drinking (ref: no) | 0.09 (-0.35, 0.53) | 0.10 (-0.42, 0.63) | 0.00 (-0.43, 0.42) | 0.20 (-0.28, 0.67) | -0.64 (-1.25, -0.03) | -0.43 (-1.53, 0.67) |
| Cigarette use (ref: never) |  |  |  |  |  |  |
| Former | 0.63 (0.01, 1.25) | 0.58 (-0.18, 1.34) | 0.53 (-0.01, 1.07) | 0.33 (-0.25, 0.91) | 1.14 (0.19, 2.10) | 1.02 (-0.39, 2.44) |
| Current | 0.04 (-0.76, 0.85) | 0.22 (-1.02, 1.47) | -0.07 (-1.01, 0.86) | 0.39 (-0.51, 1.29) | 0.05 (-0.96, 1.06) | 0.00 (-1.57, 1.58) |
| Pest/herbicide use (ref: no) | -0.46 (-1.04, 0.12) | -0.31 (-1.05, 0.42) | -0.50 (-1.04, 0.04) | -0.31 (-0.86, 0.24) | -0.14 (-0.91, 0.63) | -0.04 (-1.35, 1.28) |
| **Occupational** |  |  |  |  |  |  |
| Employed ≥ 35 hrs/week (ref: work < 35 hrs/week) | -0.07 (-0.60, 0.46) | -0.27 (-1.00, 0.45) | -0.19 (-0.75, 0.38) | 0.01 (-0.55, 0.57) | -0.15 (-1.40, 1.11) | -0.77 (-2.72, 1.17) |
| **Fire service** |  |  |  |  |  |  |
| Years of vol. FF experience (10 yrs) | -0.02 (-0.31, 0.28) | -0.03 (-0.47, 0.40) | -0.05 (-0.37, 0.27) | -0.11 (-0.57, 0.35) | -0.09 (-1.10, 0.93) | -0.01 (-1.20, 1.19) |
| Ever career FF (ref: no) | -0.72 (-1.40, -0.03) | -0.32 (-0.98, 0.34) | -0.41 (-1.02, 0.20) | -0.24 (-0.82, 0.34) | -0.07 (-1.04, 0.91) | -0.72 (-1.99, 0.55) |
| Monthly calls (ref:< 5) |  |  |  |  |  |  |
| 5 - 14 calls | 0.86 (0.28, 1.45) | 0.75 (0.02, 1.48) | 0.78 (0.22, 1.34) | 0.75 (0.13, 1.37) | 0.10 (-0.75, 0.95) | 0.52 (-0.61, 1.65) |
| ≥ 15 calls | 0.44 (-0.14, 1.02) | 1.20 (0.35, 2.05) | 0.68 (0.13, 1.24) | 0.46 (-0.12, 1.03) | -0.13 (-1.84, 1.57) | -0.69 (-2.70, 1.31) |
| Notes. Beta estimates and 95% confidence intervals (CIs) represent the change in ln-transformed PFAS per change in the independent variable, adjusted for the other factors shown and accounting for clustering within fire departments. CAPS: Cancer Assessment and Prevention Study; FF: firefighter. | | | | | | |
